# Dynamic modelling of the mTOR signalling network reveals complex emergent behaviours conferred by DEPTOR

**DOI:** 10.1038/s41598-017-18400-z

**Published:** 2018-01-12

**Authors:** Thawfeek M. Varusai, Lan K. Nguyen

**Affiliations:** 1 European Bioinformatics Institute, EMBL-EBI, Wellcome Genome Campus, Hinxton, Cambridgeshire CB10 1SD UK; 2 Department of Biochemistry and Molecular Biology, Monash University, Melbourne, Victoria 3800 Australia; 3 Biomedicine Discovery Institute, Monash University, Melbourne, Victoria 3800 Australia; 4 Systems Biology Ireland, Conway Institute, University College Dublin, Belfield, Dublin 4, Ireland

## Abstract

The mechanistic Target of Rapamycin (mTOR) signalling network is an evolutionarily conserved network that controls key cellular processes, including cell growth and metabolism. Consisting of the major kinase complexes mTOR Complex 1 and 2 (mTORC1/2), the mTOR network harbours complex interactions and feedback loops. The DEP domain-containing mTOR-interacting protein (DEPTOR) was recently identified as an endogenous inhibitor of both mTORC1 and 2 through direct interactions, and is in turn degraded by mTORC1/2, adding an extra layer of complexity to the mTOR network. Yet, the dynamic properties of the DEPTOR-mTOR network and the roles of DEPTOR in coordinating mTORC1/2 activation dynamics have not been characterised. Using computational modelling, systems analysis and dynamic simulations we show that DEPTOR confers remarkably rich and complex dynamic behaviours to mTOR signalling, including abrupt, bistable switches, oscillations and co-existing bistable/oscillatory responses. Transitions between these distinct modes of behaviour are enabled by modulating DEPTOR expression alone. We characterise the governing conditions for the observed dynamics by elucidating the network in its vast multi-dimensional parameter space, and develop strategies to identify core network design motifs underlying these dynamics. Our findings provide new systems-level insights into the complexity of mTOR signalling contributed by DEPTOR.

## Introduction

Discovered in the early 1990s as an anti-fungal agent produced by the soil bacterium *Streptomyces hygroscopicus*, rapamycin has continually surprised scientists with its diverse clinical effects including potent immunosuppressive and anti-tumorigenic properties^1–3^. It took almost two decades until the *in vivo* target of rapamycin was identified in yeast, named ‘Target of Rapamycin’ (TOR), which is a well-conserved serine/threonine kinase^4^. Nowadays, the signalling network centred on the mechanistic TOR homolog (mTOR) is known to be a complex network that plays pivotal roles in controlling cell growth and metabolism through sensing, integrating and responding to a variety of environmental cues^5^. Deregulation of the mTOR signalling network underlies many human diseases including cancer, diabetes and neurological disorders^5^. Thus, gaining a systems-level understanding of the mTOR network is critical in the development of improved treatment to these diseases.

mTOR belongs to the phosphoinositide 3-kinase (PI3K)-related kinase family. It interacts with several proteins to form two physically and functionally distinct complexes named mTOR complex 1 (mTORC1) and 2 (mTORC2), both having kinase activity. mTORC1 and 2 share common subunit proteins (e.g. mLST8, Tti1/Tel2 complex, DEPTOR)^6^ but also possess their own components (e.g. Raptor, PRAS40 for mTORC1 and Rictor, mSin1, protor1/2 for mTORC2). The exclusive binding partners are believed to determine the substrate specificity and thereby underlie the specific functions of mTORC1 and mTORC2^7^. However, how the shared or distinct subunit proteins function to coordinate the differential activities of mTORC1/2 are poorly understood.

The complexity of the mTOR signalling network has rapidly expanded over the past decade with discoveries of new components and regulatory mechanisms^8^. DEPTOR (DEP-domain containing mTOR-interacting protein) was recently identified as an mTOR-interacting protein and component of both mTORC1 and 2^9^. Importantly, DEPTOR binds to mTOR and endogenously inhibits the kinase activities of both mTORC1 and 2. Subsequent studies revealed that upon activation by growth factors and other upstream signals, mTORC1/2 phosphorylate DEPTOR and facilitate its recognition by the F-box protein βTrCP E3 ligase, triggering ubiquitination and ensuing proteosomal degradation^10–12^. Thus, mTORC1 and 2 directly regulate DEPTOR expression through controlling its protein stability. These data together demonstrate that DEPTOR and mTORC1/2 reciprocally inhibit each other, generating double-negative feedback loops. In addition, the sharing of DEPTOR by mTORC1 and mTORC2 suggests possible competition of these complexes for DEPTOR that may elicit functional consequences when DEPTOR level is limited. However, how these feedback mechanisms, protein competition and various post-translational modifications (PTMs) interplay to regulate mTOR signalling, and how DEPTOR coordinates mTORC1/2 activation dynamics have not been characterized.

The identification of DEPTOR as a direct mTOR inhibitor has led to a wave of studies investigating its role in cancer development and progression^13^. Consistent with its inhibitory effect on mTORC1/2 (often activated in cancer), DEPTOR is frequently down-regulated in most tumours^14^. However, DEPTOR is also found highly expressed in a subset of multiple myeloma, thyroid carcinoma and lung cancer^9,13^. Increased DEPTOR was thought to relieve the negative feedback from mTORC1 to IRS1 and thus activate the PI3K/Akt signalling axis, driving oncogenesis. These observations not only implies DEPTOR as a promising therapeutic target, they also point to a possible dual role of DEPTOR in cancer cells that is likely context specific. Understanding DEPTOR functions at a network level will illuminate its context-dependent properties.

In this paper, we investigate the emergent dynamic properties of the mTOR signalling network and examine how DEPTOR controls network behaviours in different settings. Using computational modelling to elucidate these dynamics, we demonstrate that the DEPTOR-mTORC1/2 network can display a wide range of highly non-linear dynamics, including bistable, multi-bistable and oscillatory behaviours. Importantly, the system can transition between distinct dynamical regimes through modulating single factors such as DEPTOR protein expression. Our findings unveil the intrinsic complexity of the mTORC1/2 activity dynamics enabled by DEPTOR, and allow for direct experimental testing. Given the important role of mTOR signalling in cancer, our findings provide new insights that will facilitate the development of intervention strategies targeting this signalling network.

## Model

### Key experimental observations and kinetic model building

**Figure 1:**
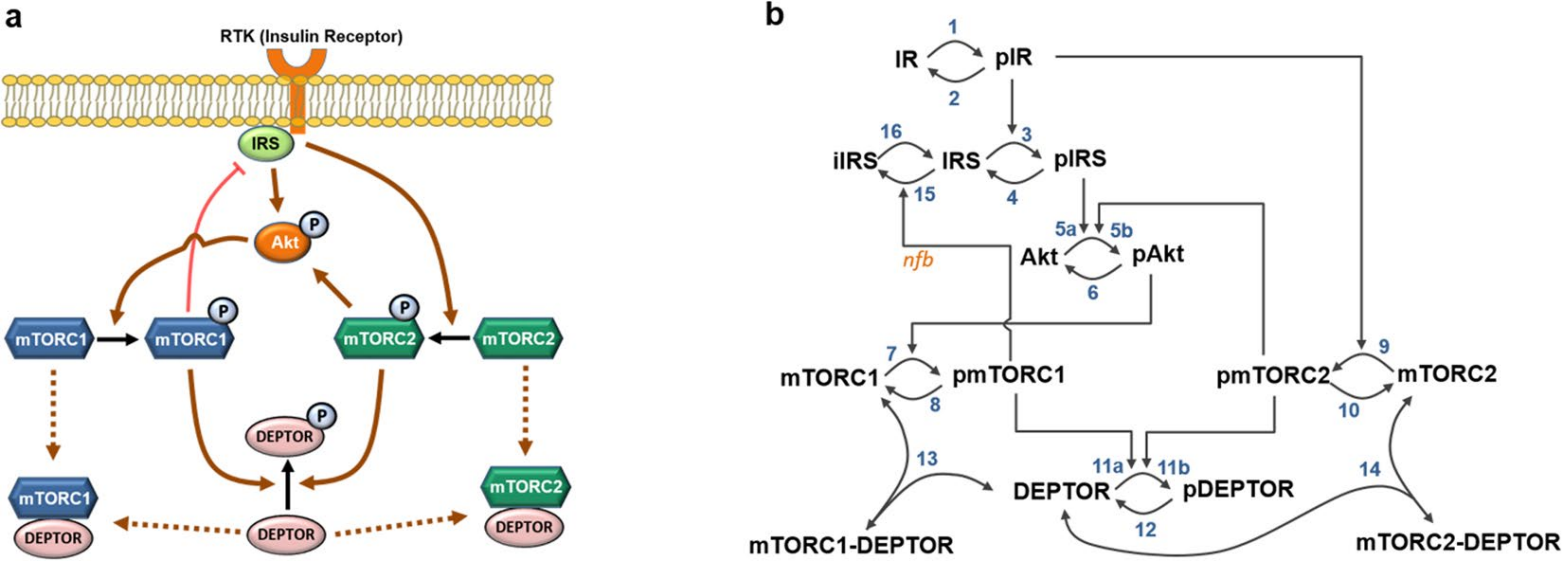
Kinetic schemes of the DEPTOR-mTOR signalling network and mathematical model. (**a**) Simplified diagram depicting the interactions and feedback loops within the DEPTOR-mTOR network. Normal, blunt and dashed arrows indicate positive, negative regulations and complex formation, respectively. (**b**) Detailed reaction scheme used to construct the DEPTOR-mTOR mathematical model (here DEPTOR synthesis and degradation are neglected on short timescales (<2hrs), see Fig. S4 for the long-timescale model). The reactions are numbered for ease of reference and described in details in the main text and Tables S1, 2, Supplementary Information (SI). The prefix “p” denotes phosphorylated (active) proteins (e.g. pmTORC1/2) and “i” denotes inactive proteins (e.g. iIRS). RTK = Receptor Tyrosine Kinase, IR = Insulin Receptor, IRS = Insulin Receptor Substrate, mTOR = mechanistic Target of Rapamycin, mTORC1/2 = mTOR Complex 1/2, PI3K = Phosphoinositide 3-Kinase, DEPTOR = DEP domain-containing mTOR-interacting protein.

DEPTOR-mTORC1/2′s mutual interactions are embedded within a larger network consisting of the PI3K/Akt/mTORC1 and mTORC2 signalling pathways. We developed a kinetic model that encapsulates the salient molecular interactions within this network, based on careful examination of existing biological data. The model is formulated using ordinary differential equations (ODEs), where the reactions are described using a combination of mass action and Michaelis-Menten kinetic laws. We modelled the system on two different timescales: (i) a short timescale (<2 hours) where biochemical events (such as phosphorylations and protein-protein interactions) preceding proteosomal degradation take place but degradation reactions can be neglected as the protein abundances in the system have not yet significantly changed (we term this the “closed model”, i.e. the conservation laws apply, Fig. 1b); and (ii) a long timescale (>2 hours) where protein synthesis and degradation are explicitly modelled (the “open model”, see Fig. S4a). Importantly, we show that inclusion of protein synthesis/degradation does not practically change the network dynamics observed at the shorter timescale. Below, we describe the key molecular mechanisms and assumptions based on which the models were built.

#### Activation of the PI3K/Akt/mTORC1 cascade

The PI3K/Akt/mTORC1 signalling cascade is activated and converged upon by a variety of Receptor Tyrosine Kinases (RTKs), most notably the Insulin Receptor (IR)^15,16^. Activation of the IR (reaction 1, which is opposed by the dephosphorylation reaction 2, depicted in Fig. 1b) leads to phosphorylation and activation of the Insulin Receptor Substrate 1 (IRS1, reactions 3-4). Once IRS1 is activated, PI3K binds to the receptor-bound active IRS1 and is phosphorylated. This activates PI3K which in turn phosphorylates membrane-bound PIP2 to PIP3, and recruits Akt to the membrane where Akt is phosphorylated at threonine 308 (T308) and activated by phosphoinositide-dependent kinase 1 (PDK1). For simplicity, we neglect the intermediate reactions in the IRS/PI3K/Akt activation cascade and model Akt activation directly by active IRS1. This event is described by reaction 5a in Fig. 1b. Importantly, Akt is also one of the best known substrates of mTORC2. Active mTORC2 phosphorylates Akt at serine 473 (S473) which further contributes to Akt activation^17^ (reaction 5b, Fig. 1b). Experimental studies suggest that phosphorylations of the two sites can occur independently, and Akt is fully activated when both are present^17,18^. However, Akt phosphorylated at either site is able to display kinase activity^19^. Thus, we modelled Akt activation as an OR gate where either PDK1 or mTORC2 can trigger Akt activation and their effects are additive.

#### Activation of mTORC1

Upon activation, Akt phosphorylates the tuberous sclerosis 1 (TSC1)-TSC2 complex, thereby suppressing the GTPase activating protein (GAP) activity of TSC1–TSC2 towards Rheb (Ras homologue enriched in brain)^20^, a positive regulator of mTORC1^21^. Thus, Akt activates mTORC1 by inhibiting TSC1–TSC2 and triggering Rheb activation. Reaction 7 in Fig. 1b denotes mTORC1 activation as a single phosphorylation event catalysed by active Akt, while dephosphorylation of mTORC1 is described by reaction 8.

#### Activation of mTORC2

Unlike mTORC1, the upstream activators of mTORC2 are not well defined. Yet, growth factors including insulin are known to trigger mTORC2 activity^22^. Whether PI3K is involved in the activation process of mTORC2 remains controversial^23–25^, and if true the exact molecular mechanism still remains obscure. Since the mechanistic details of mTORC2 activation are unknown, we assume that the activated receptor can trigger mTORC2 activation (reaction 9).

#### DEPTOR inhibits mTORC1 and 2

DEPTOR protein has two DEP domains and one PDZ domain. It is reported to bind with mTOR in the inactive forms of mTORC1 and mTORC2 through the PDZ domain. This interaction results in the inhibition of kinase activity of the mTOR complexes^9^. In our model, we assume that DEPTOR inhibits the activity of mTORC1/2 by sequestering the inactive forms of the complexes, described by the association/dissociation reactions 13-14 in Fig. 1b.

#### Active mTORC1/2 inhibit DEPTOR

In the active state, mTORC1 and mTORC2 phosphorylate DEPTOR at S293 and S299 which prime DEPTOR for further phosphorylation by CK1α at S286, S287 and S291^9,12^. Hyper-phosphorylated DEPTOR binds to βTrCp (beta-transducin repeat-containing E3 ubiquitin protein ligase) and is ubiquitinated by the SCF (Skp1, Cullins, F-box proteins) E3 ligase, consequently being targeted for proteosomal degradation^12^. Taken together, active mTORC1/2 phosphorylate DEPTOR and target it for degradation. Our model lumps the cascade of DEPTOR phosphorylation events into a single reaction for simplicity (reactions 11, Fig. 1b), which is a reasonable assumption to keep the model simple without compromising the salient network dynamics^26^. Furthermore, to ensure that the network does eventually reaches steady states, we assumed that DEPTOR phosphorylation is reversible and the opposing dephosphorylation reaction is catalysed by a general, unknown phosphatase (reaction 12, Fig. 1b), which is implicitly described by the maximal velocity parameter (V_12_, Table S1). Although specific DEPTOR phosphatase(s) have not yet been reported, they are likely to exist in order to prevent an excessive build-up of phosphorylated DEPTOR in the cells. This assumption is further justified as phosphorylation events typically take place in a much faster timescale than degradation processes. In the closed model, phosphorylated DEPTOR represents a pool of DEPTOR moiety that could not bind mTOR to inhibit the mTOR complexes, whereas in the open model phosphorylated DEPTOR is explicitly degraded, balanced by DEPTOR synthesis^27^. As mTORC1 and 2 independently phosphorylate DEPTOR, DEPTOR phosphorylation can be catalysed by either mTORC1 or 2 (reactions 11a,b). DEPTOR dephosphorylation by implicit phosphatases is described by reaction 12 (Fig. 1b).

#### Negative feedback loop to IRS1 mediated by mTORC1 and downstream signals

Upon activation, mTORC1 activates p70S6 kinase (S6K1), a key mTORC1 substrate, which in turn phosphorylates IRS1 at multiple serine residues that disrupt IRS1 activity^28–31^. Other studies also report mTORC1 can also directly inhibit IRS1 by phosphorylation^32^, or inhibits the IRS1-Akt axis via Grb10^33,34^. These mechanisms constitute functionally redundant negative feedback loops from mTORC1 to IRS1 either directly or indirectly. As these feedbacks act similarly from a dynamical viewpoint, we include only a single feedback emanated from mTORC1 (reaction 15) in the model, where mTORC1 catalyses the conversion of IRS1 to its inactive form (iIRS) by phosphorylation, which can be dephosphorylated by reaction 16.

#### Kinetic equations and model implementation

The model ODEs, rate equations and ‘nominal’ parameter values used for simulations are given in the Supplementary Information (SI). As our main objective is to characterize possible emergent properties of the DEPTOR-mTOR network under diverse physiological settings, we aim to explore the network behaviour over wide ranges of kinetic parameters rather than constraining them to a specific dataset from a particular experimental model. However, model parameters are constrained by biologically plausible values^26,35^. Specifically, the rates of protein-protein interactions are given by mass-action (MA) law, and that of (de)phosphorylation reactions are given by Michaelis-Menten (MM) law, often used to describe enzymatic reactions^27,36,37^. Protein dissociation constants in binding events typically lie in the low nanomolar range for strong bonds and in the low micromolar range for weak bonds. The k_on_ (association) rates are limited by the rate of collisions, which is limited by the rate of diffusion approximately ranging from 0.1 to 10 nM^−1^s^−1^ ^38^. Michaelis-Menten constants (K_m_) typically vary over a broad range and to explore a wide parameter space, they range from 1 to 1000 (nM). Catalytic constants (k_c_) are set between 0.0001 to 1 (s^−1^) and the maximal velocities (V_m_) from 0.001 to 10 nMs^−1^. The models were implemented and simulated using Wolfram Mathematica^39^; and bifurcation and dynamical analyses were conducted using XPPAUT^40^ and DYVIPAC^41^ (see SI for more details).

### Brief review of existing models

The PI3K/Akt/mTOR pathway has been considered for modelling by previous studies. In an early effort, Araujo RP *et al*. constructed a simplistic ODE model of mTOR signalling, but their assumption that Akt positively regulates IRS1 was not experimentally supported^42^. Around the same time, Kuepfer *et al*. studied TOR signalling in budding yeast using an ensemble of ODE models^43^. In 2009, Jain *et al*.^44^ published a quantitative model of the mTOR signalling network in the context of memory formation, evaluating the possibility of bistability in protein synthesis. Vinod *et al*.^45^ published an ODE based model focussing on the crosstalk between amino acids/nutrients and insulin signalling to investigate their roles in regulating tumour growth and insulin resistance. More recently, Pezze *et al*. developed a dynamic model that includes both mTORC1 and mTORC2^46^, which suggests that mTORC2 is activated by a pool of PI3K not involved in the negative feedback loop from mTORC1/S6K to IRS1, which is consistent with our model assumptions. However, DEPTOR has not been considered in any of these models. This study thus represents the first model of the DEPTOR-mTOR interaction network.

## Results

### Complex and emergent dynamics of the DEPTOR-mTOR signalling network

Although the presence of feedback mechanisms is indicative of nonlinear behaviours, the emergent dynamic properties of the DEPTOR-mTOR network has not been characterized. To probe the range of possible dynamics exhibited by this system, we carried out large-scale simulations where model kinetic parameters were allowed to freely and simultaneously vary over their physiological ranges, revealing the system could indeed display a variety of distinct complex behaviours that can be exploited by cells to modulate mTORC1/2 activities and cellular responses, including bistable switches and sustained oscillations. Here, we describe the salient observed network behaviours and analyse how they are regulated.

#### The mTOR network shifts between distinct and complex dynamical regimes

**Figure 2:**
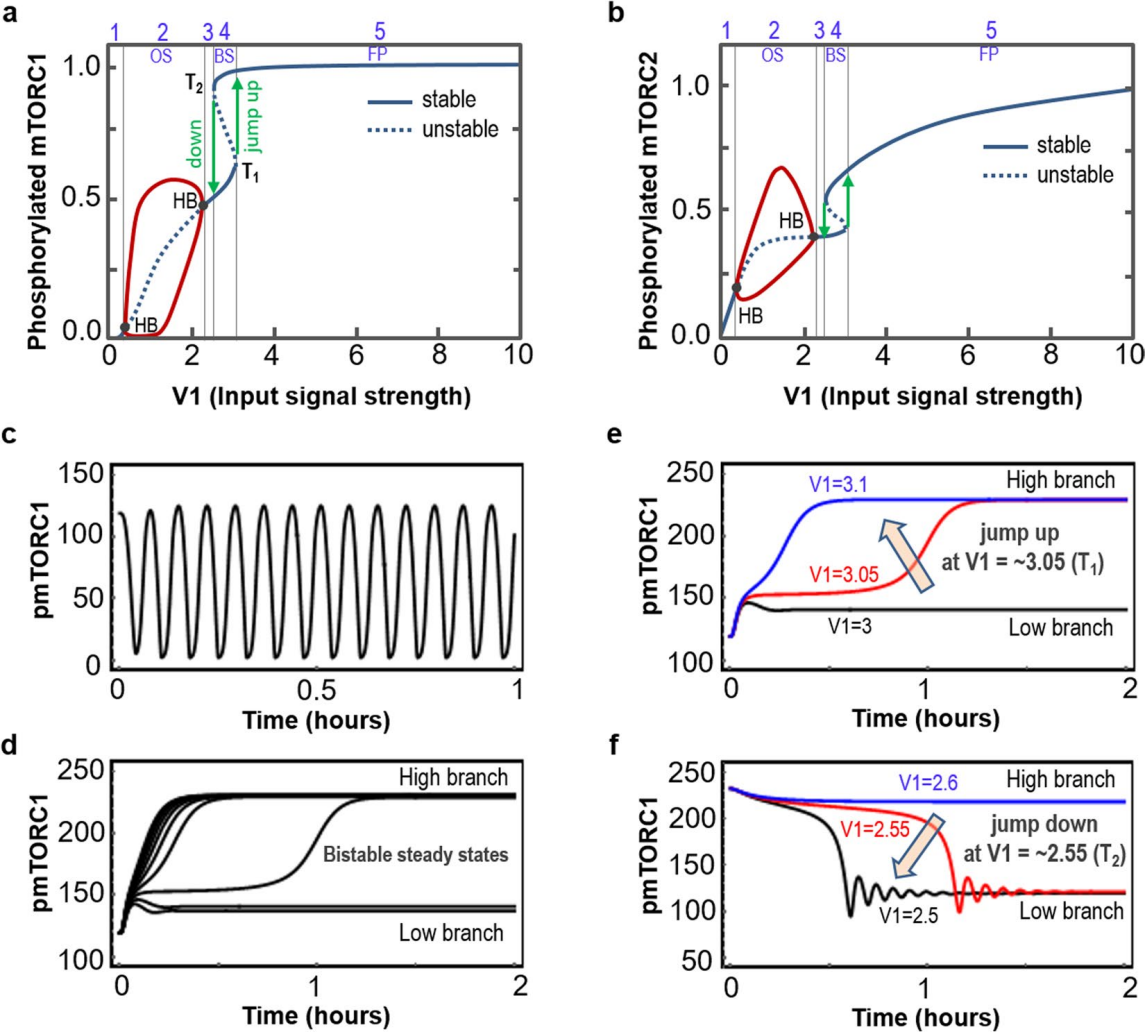
Oscillation, bistability and hysteresis in the DEPTOR-mTOR network. (**a**,**b**) Dependence of the steady-state levels of (normalised) phoshorylated mTORC1/2 on increasing strength of the input signal (represented by parameter V_1_, see Table S1). Stable (unstable) states are shown in solid (dotted) lines. HBs indicate Hopf bifurcation, and the turning points T_1_ and T_2_ indcate saddle-node bifurcations. The dynamic regions are numbered from 1 to 5 where regions 1, 3 and 5 display fixed-point (FP), region 3 displays oscillation (OS), and region 4 displays bistability (BS) dynamics. Parameter values used are given in Table S1 and Table S2. (**c**) Oscillatory temporal dynamics of pmTORC1, V_1_ = 1. (**d**) Bistable steady states of pmTORC1 showing the high and low braches can both be reached by different initial conditions when the system resides within the BS regime, V_1_ = 3. (**e**) Starting at the LOW steady state branch, temporal simulation shows pmTORC1 jumps to the HIGH branch when V_1_ is increased. (**f**) Starting at the HIGH steady state branch, temporal simulation shows pmTORC1 jumps to the LOW branch at a different threshold when V_1_ is decreased. The remaining parameter values used are given in Table S1 and Table S2.

As insulin is secreted by beta cells of the human pancreas in response to blood glucose level (which is in turn determined by factors including time after meals or fasting), the ambient plasma insulin level can fluctuate within a wide physiological range on a daily basis^47,48^. We thus asked how the mTOR system responds to changes in IR activation induced by insulin. Interestingly, model simulations show that under certain parameter regimes, gradual increase of a single parameter representing the rate of IR activation (V_1_) can dramatically shift the mTOR system between entirely distinct dynamical regimes. Illustrated in Fig. 2a and b for phosphorylated (active) mTORC1 and 2 as outputs, respectively; as V_1_ is increased the system transitions from a monostable, fixed-point (FP) regime (denoted R1) to a sustained oscillatory (OS) regime (R2), back to a FP regime (R3), then to a bistable (BS) regime (R4) and finally to a FP regime (R5). The oscillatory region R2 is separated from the neighbouring FP regimes by two Hopf Bifurcation (HB) points, defined as the local birth or death of a periodic solution from equilibrium as a parameter crosses a critical value^49^. In the oscillatory regime, the system cannot reside in a steady state but oscillates in a self-perpetuating manner with constant frequency and amplitude (Fig. 2c). Figure 2a further shows that the oscillation amplitude for pmTORC1/2 peak at intermediate values of V_1_ within the oscillatory range (purple lines, R2) and gradually reduces as V_1_ approaches the HB points.

Further increase of V_1_ shifts the system into a bistable regime R4, separated from R2 by a narrow FP regime (R3, Fig. 2a,b). A bistable system can switch between two distinct stable steady states but cannot settle in an intermediate (unstable, indicated by dashed lines in Fig. 2a,b) state, illustrated by simulated time-course of pmTORC1 in Fig. 2d. Bistability is one of the most common design motifs that often underlies switching behaviour in biological networks^27,50–54^. A hallmark feature of a bistable system is the hysteresis effect, which implies that the stimulus must exceed a certain threshold for the system to switch to a different steady state at which the system will resides even if the stimulus decreases past the threshold, depicted in Fig. 2a,b. As V_1_ increases towards R4, active mTORC1/2 traverse the low steady-state branches before abruptly jumping “on” to the high branches at the threshold T_1_, whereas if starting from the high steady states, mTORC1/2 activities traverse the high steady-state branches as V_1_ decreases and only jump “down” to the previous branch at a lower threshold T_2_. As the system still “remembers” its previous state even when the stimulus (V_1_) passes its original switching threshold, hysteresis is often associated with so-called “biological memory” of signalling networks^55^. The history-dependent switching thresholds and jumps are further demonstrated by the temporal simulations of pmTORC1 when V_1_ is varied around the HB points (Fig. 2e,f).

The observed bistable switches and oscillations not only occur for mTORC1/2 activities but also manifest at the level of Akt activation (Fig. S5). Given the important roles of these kinases in regulating fundamental cellular processes including protein synthesis, cell survival and autophagy; the diverse dynamics displayed by the mTOR network may enable cells to swiftly adapt to fluctuating environments through modulating just a single input. Furthermore, hysteresis-induced switches could provide robust mechanisms for cells to unambiguously turn on/off key signalling kinases by filtering out intrinsic molecular noises, thereby contributing to robust cell-fate decision making.

#### DEPTOR critically regulates mTORC and 2 activation dynamics

**Figure 3:**
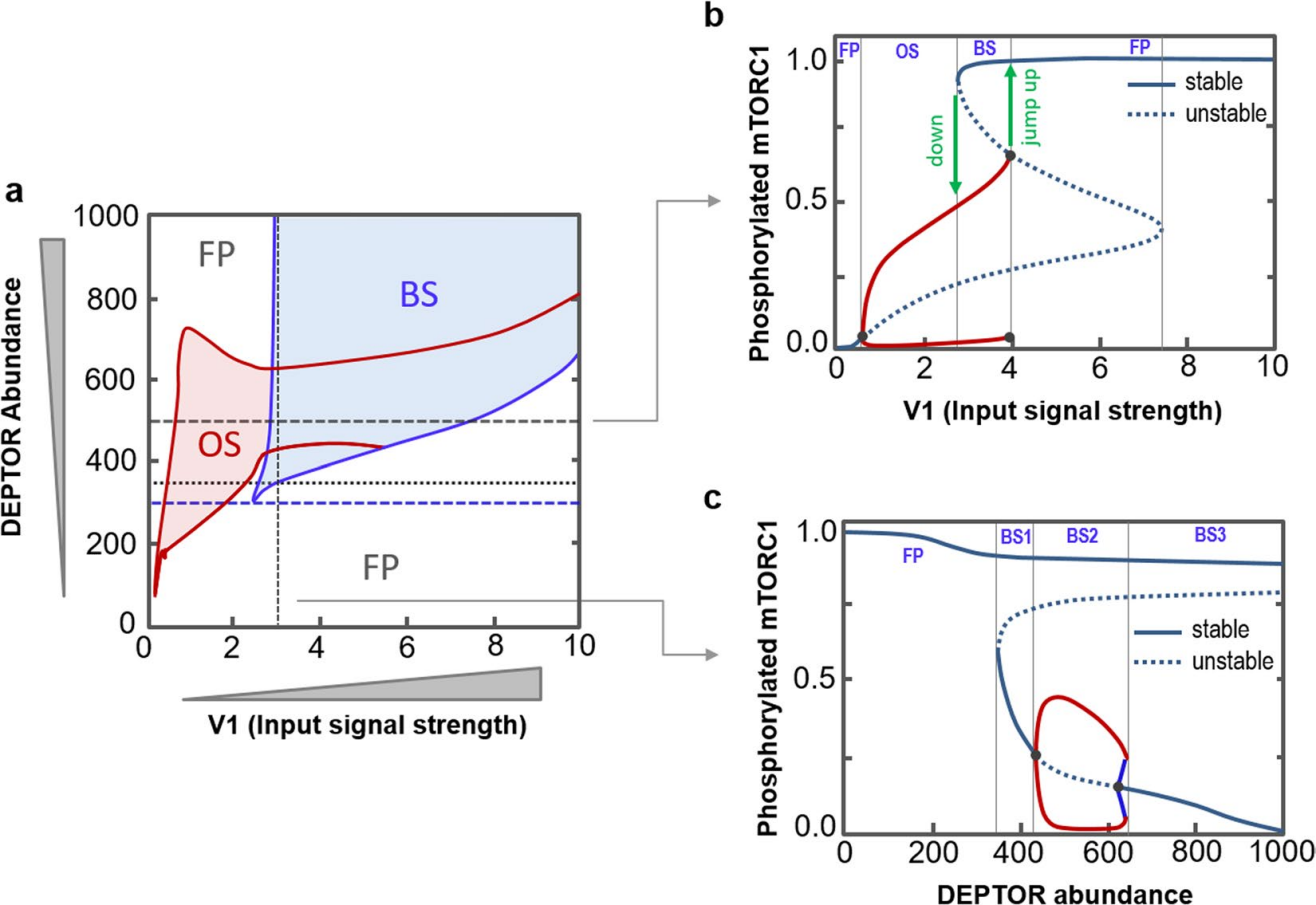
Partition of the two-dimensional parameter space into qualitatively distinct behaviours. (**a**) For different combined levels of DEPTOR abundance and V_1_, the parameter space is divided into different dynamical regimes using XPPAUT. The dashed /dotted lines show how the increase in one parameter at fixed values of the other parameter can bring different dynamics, as shown in panels b,c. (**b**) Dependence of steady-state pmTORC1 on increasing V_1_ at DEPTOR abundance = 500 (horizontal dashed line in a). (**c**) Dependence of steady-state pmTORC1 on increasing DEPTOR abundance at V_1_ = 3 (vertical dashed line in a). The remaining parameter values used are given in Table S1 and Table S2. The horizontal dotted line in panel a corresponds to the one-parameter bifurcation diagrams in Fig. 2a,b; and the blue dashed line corresponds to the bifurcation diagrams where only OS is present (Fig. S5).

As DEPTOR directly binds mTOR and mutually inhibits both mTORC1/2, we hypothesized DEPTOR exerts important influence on the activation dynamics of these complexes. To test this, we carried out two-dimensional (2D) bifurcation analysis, which reveals the dependence of the systems dynamics on simultaneous changes of DEPTOR protein abundance and V_1_ (Fig. 3a). Figure 3a partitions the parameter coordinate into different dynamical regimes previously observed, including OS, BS and FP. Interestingly, BS only occurs when DEPTOR is sufficiently abundant (restricted by a lower bound of DEPTOR level, dashed blue line); while oscillation only occur over an intermediate range of DEPTOR (restricted by lower and upper bounds, solid purple lines) regardless of V_1_. As sufficient DEPTOR is probably needed to impose functional inhibition on the mTOR complexes, these results are in line with the expectation that bistability requires DEPTOR-mediated feedback with mTOR.

Interestingly, we observe an overlapping region where OS and BS can co-exist (Fig. 3a). This co-existence is clearly shown in the one-dimensional (1D) bifurcation plot (Fig. 3b) when DEPTOR level is set at a specific value (dashed black line in Fig. 3a). Unlike in Fig. 2a,b (where DEPTOR level is set at a lower value, black dotted line in Fig. 3a), in this case increasing V_1_ moves the system from a classical OS regime into an atypical bistable regime where the system can switch between a fixed point (higher branch) and an oscillatory (lower branch) steady state (Fig. 3b). As similarly done in Fig. 2e,f, our time-course simulations revealed the switching thresholds between these steady-state branches (green lines, Fig. 3b), which appear different compared to those observed in Fig. 2a,b.

To further probe the role of DEPTOR, we directly simulate the effect of changes in DEPTOR level on the systems dynamics at various values of V_1_. Notably, Fig. 3c (and Fig. S1) shows that under specific parameter conditions (vertical dashed line in Fig. 3a), increasing DEPTOR shifts the system from a FP to a series of distinct BS regimes (BS1-3) that are characterised by co-existence of either two fixed-point stable steady states (BS1,3) or a fixed-point state and an oscillatory one (BS2). Together, these analyses show that DEPTOR confers extremely complex dynamic behaviours to the mTOR network, and DEPTOR level critically controls the dynamics of mTOR complexes activation.

### Regulation of complex network dynamics

#### Network dynamics is governed by a delicate balance of DEPTOR and mTORC1/2 abundances

**Figure 4:**
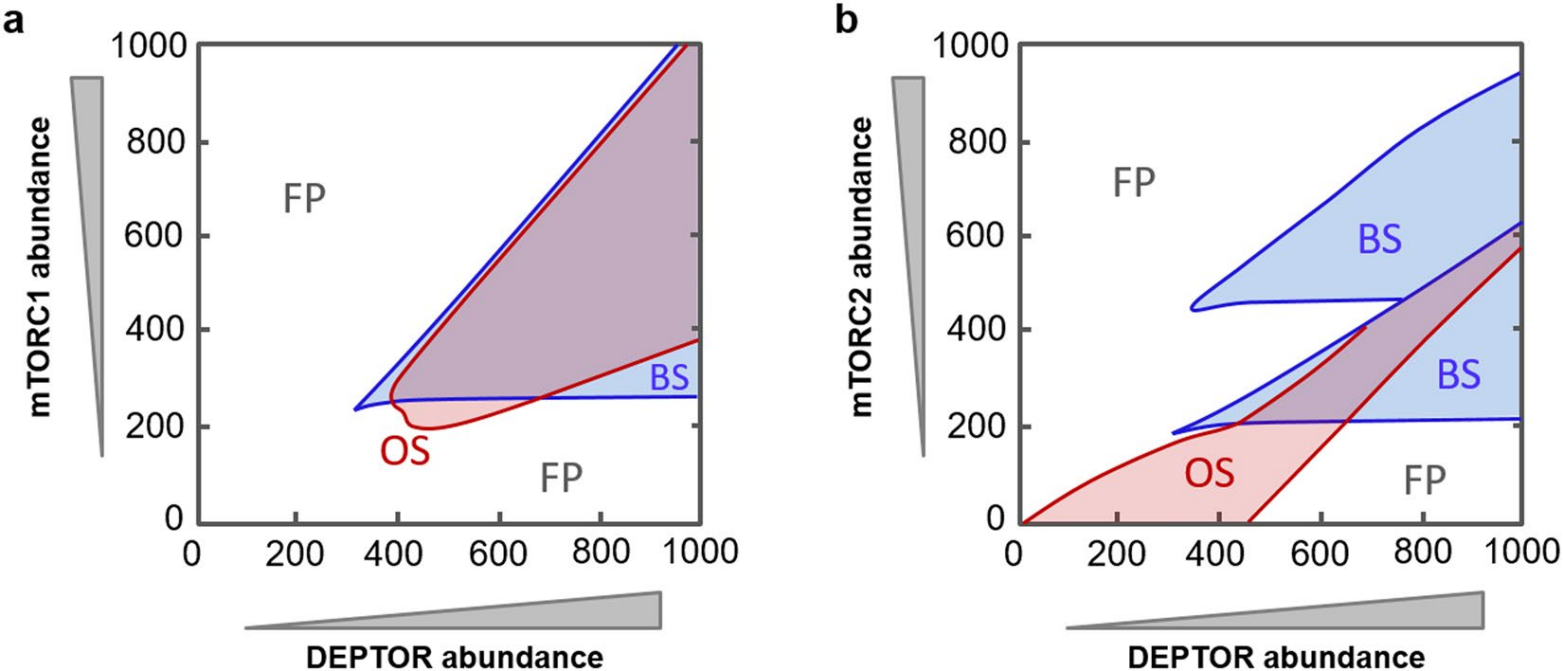
Control of dynamic behaviours by expression of DEPTOR, mTORC1 and mTORC2. For different combined levels of DEPTOR and mTORC1 (**a**) or mTORC2 (**b**), the parameter space is partitioned into qualitatively distinct behaviours. V_1_ = 2.5 and the remaining parameter values used are given in Table S1 and Table S2.

We hypothesized that the double-negative feedback mechanisms between DEPTOR and mTORC1/2 underline the emergence of bistability and contributes to the regulation of oscillations. Yet, it remains unclear whether these feedbacks and the roles of the mTOR complexes are redundant or not. We thus investigated the dependence of systems dynamics on combined changes in the expression of the mTOR complexes and DEPTOR. Interestingly, we found that while mTORC1 is required for both bistability and oscillation (Fig. 4a); mTORC2 is dispensable for oscillation, evident by the absence of a lower bound of mTORC2 for this regime (Fig. 4b). Furthermore, absence of both mTORC1/2 abolishes bistability, suggesting at least one feedback loop induced by either mTORC1 or 2 with DEPTOR is necessary for bistable switches. Moreover, the similar shapes of the BS region (Fig. 4a,b) suggest similar effects on bistability by changes of mTORC1/2 abundances. In addition, oscillation and bistability only co-exist under restricted conditions that require sufficient abundances of DEPTOR, mTORC1 and 2 (overlapping regions, Fig. 4a,b). Together, these analyses indicate that the observed complex dynamics are tightly regulated by an intricate balance between the network nodes.

#### Oscillations is brought about by the mTORC1-mediated negative feedback

**Figure 5:**
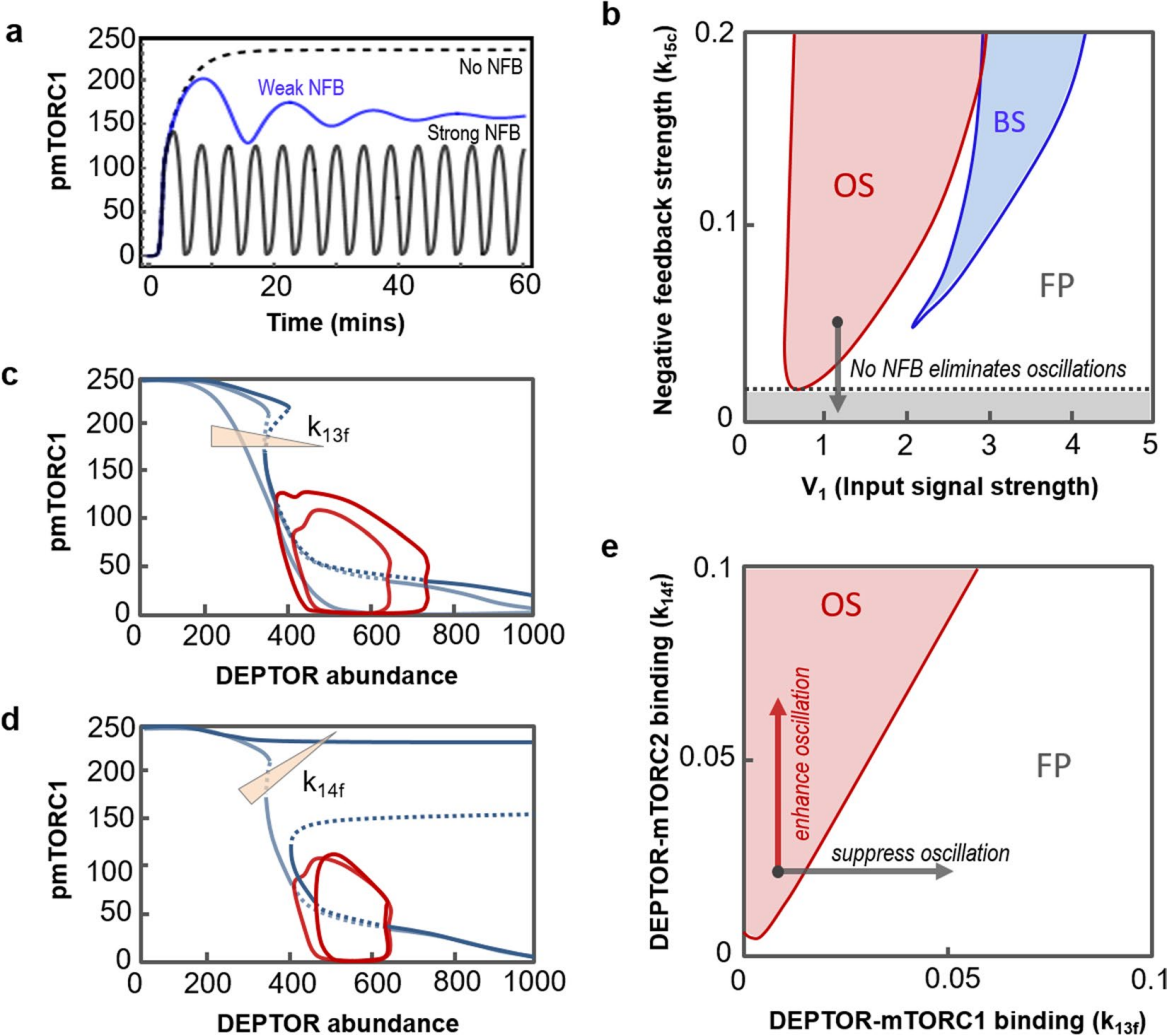
Control of dynamic behaviours by feedback loops and protein binding affinities. (**a**) Oscillation is gradually eliminated in response to decreased strength of the mTORC1-mediated negative feedback loop (k_15c_ = 0.1 (black), 0.015 (blue) and 0 (dashed line), V_1_ = 1). (**b**) 2D-bifurcation plot showing dependence of systems dynamics on combined changes of NFB strength (k_15c_) and V_1_. (**c**) 1D-bifurcation plot showing oscillation is abolished as k_13f_ increases (k_13f_ = 0.0007, 0.001 and 0.01, V_1_ = 1). (**d**) 1D-bifurcation plot showing decreasing k_14f_ (0.005 and 0.007, V_1_ = 2.5) shifts switching threshold to the right. (**e**) Dependence of systems dynamics on combined changes of DEPTOR binding affinities to mTORC1 and 2. The remaining parameter values and corresponding units are given in Table S1 and Table S2.

The inhibitory effects of mTORC1 and/or S6K towards IRS constitutes a prominent negative feedback loop in the PI3K/Akt/mTORC1 signalling axis, and has been implicated in insulin resistance^56^ as well as resistance to anti-cancer drugs^57^. Although the previous sections show that multiple factors control the size of the oscillatory regime and shape the temporal dynamics, we found that the mTORC1-mediated feedback primarily determines the existence of oscillation. Indeed, feedback interference (by perturbing reaction 15 through decreasing the catalytic rate k_15c_, Fig. 1b and SI) increasingly abolishes sustained oscillation, shown both by time-course simulations (Fig. 5a) and bifurcation analysis (Fig. 5b). Figure 5b further reveals a threshold that this feedback’s strength must exceed to enable oscillation. Importantly, complete shutting-off of this feedback (k_15c_ = 0) renders the system incapable of displaying oscillations even when we exhaustively explored the vast parameter space (not shown). In contrast, we could always find oscillation at some pockets of the parameter space so long as this feedback is present (even weak). Together, these analyses determine the mTORC1 negative feedback as the determinant of oscillation, while other mechanisms contribute to its regulation.

#### DEPTOR-mTORC1/2 complexes differentially regulate dynamic behaviours

To further examine possible differential roles of the DEPTOR-related feedback loops, we individually perturb the complex formation between DEPTOR and mTORC1 or 2 by varying their binding affinities. Surprisingly, under conditions where oscillation is predominant, stronger DEPTOR-mTORC1 binding increasingly weakens oscillations (Fig. 5c); whereas stronger DEPTOR-mTORC2 binding reinforces oscillations instead (Fig. 5d). This suggests contrasting roles of DEPTOR-induced mTOR complexes inhibition in regulating oscillatory dynamics, which could be explained as follows. A strong DEPTOR-mTORC1 association effectively sequesters mTORC1 away from its active pool, thereby weakening the mTORC1-to-IRS1 negative feedback which is the major inducer of oscillations as shown above. On the other hand, a strong DEPTOR-mTORC2 association sequesters DEPTOR from binding mTORC1, thereby releasing more mTORC1 to be activated and thus strengthening the mTORC1-to-IRS1 negative feedback, leading to more pronounced oscillations. The opposing roles of DEPTOR complex formations are further evident by observing the bifurcation plane with combined changes in the binding affinities (Fig. 5e), in which an increased DEPTOR-mTORC1 association shifts the system into the monostable (FP) region whereas increased DEPTOR-mTORC2 binding moves the system further into the oscillatory regime.

Under the parameter conditions where bistability is also present, we found that reducing DEPTOR-mTORC2 association retains bistability but shifts the switching threshold to the right, i.e. more DEPTOR is required to switch off mTORC1 (Fig. 5d). A similar trend was observed for the DEPTOR-mTORC1 association (not shown), consistent with previous observation that DEPTOR binding with mTORC1/2 similarly affect bistability. These parallel effects likely stem from the similar structure of the double-negative feedbacks between DEPTOR and mTORC1/2; whereas the differential regulation of oscillations is due to mTORC2 not being involved in the mTORC1-to-IRS1 negative feedback loop.

### Multi-dimensional analysis of network dynamics

**Figure 6:**
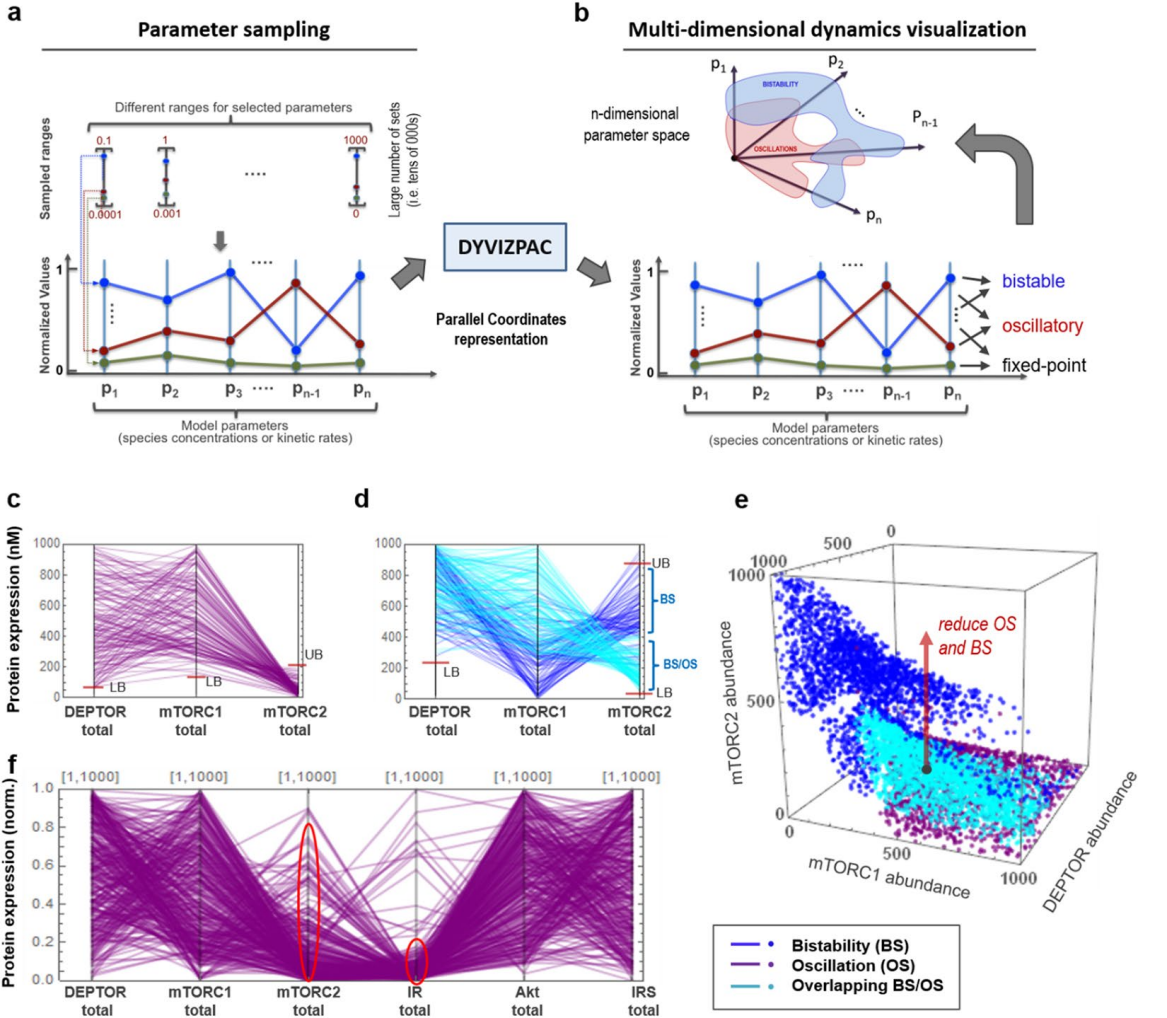
Multi-dimensional analysis and visualisation of network dynamics. (**a**,**b**) Schematic illustrating DYVIPAC-based multi-dimensional analysis of dynamic behaviours and visualisation using Parallel Coordinates (PC) plots (see Nguyen *et al*. (2015) for details). A large number of parameter sets are randomly sampled over wide ranges (**a**), and corresponding dynamics is examined and classified for each set, before being displayed on PC plots, which are effective representations of the real multi-dimensional parameter space (**b**). (**c**,**d**) A PC plot showing only the oscillatory **(c)** and bistable as well as co-existing BS/OS sets (**d**) obtained by DYVIPAC from 30,000 parameter sets where DEPTOR, mTORC1 and 2 abundances are randomly sampled within the indicated ranges. LB = lower bound, UB = upper bound. (**e**) Classical 3D plot showing all the oscillatory and bistable sets in panels c and d. (**f**) PC plot showing the oscillations-inducing sets returned from a 6D analysis where the abundances of all the model species are randomly sampled; the other dynamics are displayed in Fig. S2.

Although the 1D and 2D bifurcation analyses have yielded valuable insights into the dynamical properties of the DEPTOR-mTOR network, it is important to examine the impacts on network dynamics by combined changes of multiple parameters (i.e. >2) at the same time, thereby providing a more global understanding of network behaviours. To this end, we employ DYVIPAC we previously developed^41^ to probe and visualize network dynamics in the high-dimensional parameter space. DYVIPAC ideally complements low-dimension continuation tools such as XPPAUT, which only handle changes in two parameters or less. The workflow of DYVIPAC-based analyses is summarised in Fig. 6a,b (see^41^ for details). Briefly, the parameter space is comprehensively and exhaustively probed and each sampled parameter set is classified into specific dynamical regimes before being visualised by parallel coordinates (PC) plots which effectively reconstitute the otherwise unobservable n-dimensional “bifurcation” diagrams (particularly when n > 3) (Fig. 6b).

First, we asked how combined changes in DEPTOR and mTORC1/2 expressions impact on network dynamics. Figure 6c,d display the parameter sets (extracted out of 30,000 randomly sampled sets) at which only oscillation (purple), bistability (blue) or co-existing BS + OS (cyan) occurs (superimposed in Fig. S2a). A classical 3D representation is also given in Fig. 6e. We found that oscillations require sufficient levels of both DEPTOR and mTORC1; and favours exclusively low to no mTORC2. Furthermore, bistability requires a lower bound (LB) for DEPTOR (Fig. 6c), and both lower and upper bounds (UB) for mTORC2 (Fig. 6d). These results are in line with previous observations of 2D bifurcation plots (Fig. 4a,b). However, what is not obvious from the 2D plots is that for BS, low levels of mTORC1 tends to associate strongly with high levels of mTORC2 (blue, Fig. 6d) but for co-existing BS/OS, the opposite trend is observed (cyan). In addition, bistability exist as two separate but connected pockets within the 3D space (Fig. 6e).

We next probe the network dynamics in the 6-dimensional parameter space, where the expression of all six network components are allowed to simultaneously vary within wide ranges. Fig. 6f shows that even in this large space, oscillations still require none-to-low mTORC2, consistent with previous observations at low-dimension analyses. Interestingly, this analysis also reveals that oscillation is only robust at sufficiently low abundance of the IR (red circles, Fig. 6f). On the other hand, bistability still requires sufficiently high DEPTOR and sufficiently low mTORC1 (Fig. S2b), but appears obtainable at essentially any levels of Akt or IRS. Moreover, co-existing BS/OS occupies a rather restricted region of the 6D space (Fig. S2c). Multi-dimensional analyses also further validate the roles of feedback loops and DEPTOR-mTORC1/2 bindings observed at lower dimensions (Fig. S3).

Together, these results reconfirm the salient dynamics and regulatory features identified by low-dimension analyses but additionally reveal new global insights, providing a more comprehensive understanding of the network behaviour under vastly different parameter conditions that may manifest under very different cellular contexts.

### Core design motifs underlying complex behaviours

The above results collectively suggest that some network nodes (and links) are absolutely required for a specific dynamics, while others are dispensable but contribute to its regulation. Here, we seek to identify the core design principles within the DEPTOR-mTOR network responsible for oscillatory and bistable responses. To this end, we develop a systematic *in silico* knock-out strategy where the network node/link of interest are systematically removed and its consequence on systems dynamics is examined in the multi-parameter space using DYVIPAC. Our rationale is that complete abolishment of OS (or BS) by removal of a node/link implies its essentiality in maintaining that behaviour.

**Figure 7:**
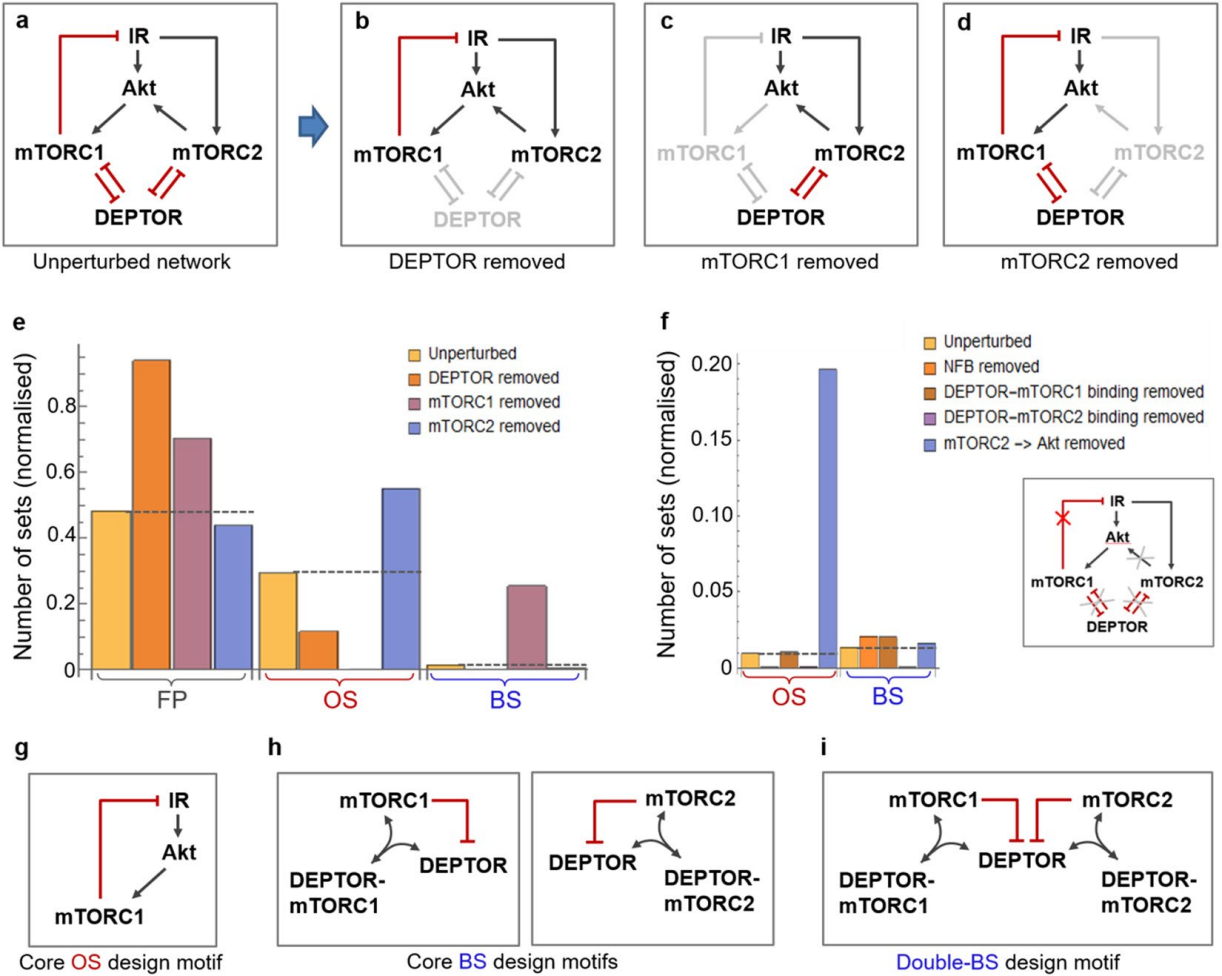
Identification of core network design motifs underlying oscillations and bistability. (**a**) An abstract interaction diagram of the intact DEPTOR-mTOR network, which is systematically perturbed by removing DEPTOR (**b**); mTORC1 (**c**); or mTORC2 (**d**) from the circuit. The removed nodes and related edges are grayed out. (**e**) Comparison of the occurrence of oscillation (OS), bistability (BS) and fixed-point (FP) dynamics between the perturbed networks and intact network (dashed lines) in panels a–d, assessed by the number of parameter sets returned by DYVIPAC surveying the 6D parameter space (as in Fig. 6f). A total of 300,000 parameter sets were randomly sampled for each network. The % number of sets is normalised between 0 and 1. (**f**) Similar as in panel e but here four key network edges were perturbed as indicated. Only the OS and BS sets are displayed. (**g–i**) The stripped-down, core network design motifs identified that are capable of generating the corresponding dynamic behaviours.

Using this strategy, we first consider perturbations where DEPTOR, mTORC1 or mTORC2 is alternatively knocked out (Fig. 7b–d) compared to the intact network (Fig. 7a). For each perturbed and the original network, 300,000 parameter sets were randomly generated in the 6D space (as in Fig. 6f) and their corresponding dynamics classified. Figure 7e shows that removal of DEPTOR or mTORC2 does not eliminate OS, but mTORC1 removal did, consistent with previous findings that mTORC1’s NFB underlines OS. Interestingly, absence of mTORC2 confers the network more robust to OS, evident by a significantly larger number of OS parameter sets in the mTORC2-null network (Fig. 7d,e). Removal of DEPTOR completely eliminates BS, suggesting its essentiality for this dynamics. Surprisingly, mTORC1 removal renders enhanced BS whereas removal of mTORC2 reduces BS, indicating either mTORC1 or 2 is sufficient to generate BS but they have antagonizing effects when both are present. This highlights that integrating multiple BS-generating motifs does not necessarily increase BS robustness.

Next, we selectively delete key network links, as shown in Fig. 7f. Compared to the original network, deletion of the NFB completely kills OS. Unlike removal of mTORC1, blocking DEPTOR-mTORC1 binding does not affect OS or BS. Intriguingly, blocking DEPTOR-mTORC2 binding significantly reduces OS, which is opposite to the effect of mTORC2 removal (Fig. 7e). This is probably because the controlling effect mTORC2 has on OS is mediated mainly via Akt activation rather than its association with DEPTOR, as removing the mTORC2-Akt link significantly boosts OS occurrence (Fig. 7f), similar to the removal of mTORC2. In contrary, mTORC2′s effect on BS is mediated via DEPTOR instead of Akt.

Together, these analyses show that mTORC1 and DEPTOR are essential for OS and BS, respectively; and mTORC2 promotes BS but inhibits OS. These results also allow us to distil the core network design motifs that underlie OS or BS (Fig. 7g,h). Interestingly, although either DEPTOR’s mutual inhibition with mTORC1 or 2 is sufficient to induce bistability, only a design combining both could give rise to double-BS dynamics seen previously (Figs 4b and 6e), where multiple distinct BS regions exist within the parameter space.

### Long-timescale model with explicit DEPTOR synthesis and degradation

In this model, the synthesis and degradation of DEPTOR are explicitly taken into account (reactions 17 &18 in model scheme given in Fig. S4a). Zhao *et al*.^10^ determined the half-life (t_1/2_) of endogenous DEPTOR to be ~6–12 hours, based on which we estimated DEPTOR’s degradation rate to be ~3 × 10^−5^ (s^−1^, degradation rate = ln(2)/ t_1/2_); while DEPTOR’s synthesis rate is assumed to be of typical value for protein production (see Table S1). Importantly, explicit inclusion of DEPTOR synthesis/degradation in this long-timescale model show that the intricate dynamic features of the DEPTOR-mTOR systems discussed above, including oscillatory and bistable behaviours, remain essentially the same on short timescales (<2 hrs). As can be seen in Fig. S4, over the first 2 hours, the long-timescale model behaves practically indistinguishable from the short-timescale (“closed”) model which neglects protein synthesis and degradation; and both oscillatory (Fig. S4b) and bistable (Fig. S4c) responses are observed. On the long timescale (»2 hrs) however, complex dynamics such as bistability and oscillations might not be exhibited due to the effect of protein degradation, and the system would approach a stable steady state (at selected parameter values, see Fig. S4). To ensure that these observations are not specific to one set of parameter, we performed similar comparative simulations for multiple parameter sets (Figs S6 and 7). As the timescale of (de)phosphorylation and protein-protein binding events in the DEPTOR-mTOR system are typically in the seconds-to-minutes timescale, which is significantly shorter that the timescale associated with DEPTOR synthesis and degradation (i.e. several hours); we have thus mainly focused on the short (2-hr) timescale over which the system’s behaviour is essentially similar to that observed for the closed model.

## Discussion

Cellular behaviours are shaped by the precise control of protein activities, which are in turn coordinated by complex signalling networks featuring intricate feedback loops and interaction events. Complex dynamic behaviours often emerge in these systems, including all-or-none or bistable switches and oscillations, which help direct cell-fate decisions. For instances, we have shown that the life/death balance in cells is in part orchestrated by the molecular switches in the Hippo/MST2-Raf-1 crosstalk network^37,51,58^; alternative gene expression programs are dictated by the bistable responses of histone H2A ubiquitination in the Ring1B/Bmi1 system^27^; and recently cell motility and actin cytoskeleton dynamics behave in an on/off manner driven by bistability in the Rac1/RhoA network^50,54^. On the other hand, synchronized oscillations are crucial for the maintenance of circadian rhythm, and periodic pulses of p53 encode information during the response to DNA damage^59–61^. However, despite the intricacies of the DEPTOR-mTOR circuitry, a detailed understanding of the network’s dynamical properties has been lacking. To address this gap, we constructed mathematical models encapsulating the interactions between DEPTOR, the PI3K/Akt/mTORC1 and mTORC2/Akt pathways and used these models to study systems dynamics in details.

Our work reveals for the first time an extremely rich repertoire of dynamical behaviours (regimes) exhibited by the DEPTOR-mTORC1/2 network. In particular, we show that alteration of even a single input such as the level of insulin stimulation or DEPTOR expression, do not merely change the response amplitude but can drastically transform the system responses. Upregulation of DEPTOR, for example, can shift the system between oscillatory, bistable and co-existing oscillation/bistable regimes, and vice versa. Notably, increasing (decreasing) DEPTOR could switch off (on) mTORC1/2 activities in abrupt switch-like manners, at different switching thresholds due to hysteresis. Moreover, while DEPTOR consistently inhibits mTORC1/2, its effects towards Akt are predicted to be highly non-monotonic. At low levels, DEPTOR inhibits Akt but at high levels, DEPTOR enhances Akt activation (Fig. S5a). These findings are compelling as DEPTOR levels have been found inconsistent and highly variable across different cancer types. It is down-regulated in most tumours^14^, but is over-expressed in multiple myeloma, thyroid carcinoma and lung cancer^9,13^. Interestingly, both down- or over-expression of DEPTOR can lead to increased cell proliferation, possibly by different mechanisms^13,14^. Our simulations confirm these observations and show that reduced DEPTOR can induce proliferation and cancer progression by releasing the inhibitory breaks on mTORC1/2, whereas upregulated DEPTOR may promote cancer by activating Akt instead. Importantly, model simulations uncover a therapeutically-relevant “expression window” of DEPTOR, within which low Akt as well as mTORC1/2 activities could be achieved (Fig. S5). If proven, further model-based analysis will be valuable in understanding how such a window could be modulated for therapeutic purposes.

In addition to cancer, our model predictions also support recent biological findings of the *in vivo* impact of DEPTOR loss or overexpression in the context of cellular metabolism, insulin resistance and obesity^62–65^. For instance, specific overexpression of DEPTOR in the mediobasal hypothalamus (MBH), a brain region regulating energy balance, as well as systemic overexpression of DEPTOR (brain and periphery) prevents high-fat diet-induced obesity, improves glucose metabolism and protect against hepatic steatosis^64^. Importantly, these phenotypes are associated with activated PI3K-Akt signalling via dampened mTORC-mediated negative feedback caused by increased level of DEPTOR^64^. This is in agreement with model predictions that high DEPTOR level inhibits mTORC1 and concomitantly upregulate Akt activity (Fig. S5a). By the same mechanism, transgenic mice overexpressing DEPTOR promotes white adipose tissue (WAT) and adipogenesis through activation of the proadipogenic Akt-PPAR-γ axis, supporting a correlation between DEPTOR expression and the degree of obesity in human^65^. Similarly, another *in vivo* study further showed that DEPTOR induced by overexpressing Baf60c in a muscle-specific transgenic mouse model could activate Akt and glycolytic metabolism, indicating a critical role of DEPTOR in the specification of fast-twitch muscle^62^. In contrast, knocking out DEPTOR specifically in the liver of mice resulted in sustained mTORC1 activity and reduced circulating glucose upon fasting^63^. Interestingly, Akt phosphorylation was not affected by DEPTOR loss, suggesting a weak mTORC1-mediated feedback in this context^63^. Our model may be able to explain these observations as reducing DEPTOR level results in a drastic switch-like increase in mTORC1 activity whereas it affects Akt activity in a much less significant manner (Fig. S5b,c). Taken together, our model of the Akt-mTOR-DEPTOR network could reconcile and explain a range of empirical findings in the field. It would be particularly interesting for future studies to tailor this generic model for specific biological and disease contexts to examine potential context-specific behaviours and formulate new testable hypotheses.

Combining low-dimensional bifurcation analyses and high-dimensional stability analyses exploring the system in the vast multi-parameter space, we have comprehensively characterised the conditions governing each of the observed complex behaviours. We show that the existence of oscillations and/or bistability are coordinated by a delicate balance between the availability of DEPTOR, mTORC1 and 2, as well as the competing bindings between DEPTOR and mTORC1/2. We show that oscillations arise primarily from the mTORC1-to-IRS negative feedback loop; whereas bistability requires the mutual inhibition between mTORC1 or 2 and DEPTOR, either mechanism is sufficient for bistability but in combination resulting in richer bistable dynamics. Both types of dynamics, however, can be fine-tuned by a broad array of factors. It is of note that unlike inhibition by post-translational modifications, DEPTOR inhibits mTORC1/2 through inhibitory bindings, constituting the double-negative feedback loops. The results reported here are in line with our previous work showing that (reversible) protein-protein bindings could trigger diverse and complex dynamic behaviours, including bistability and oscillations^52^. In complement to numerical analyses, we also carried out parameter-free network analyses of complex behaviours based on Reaction Network Theory^66,67^ using the Chemical Reaction Network Toolbox (CRNT, www.crnt.osu.edu/CRNTWin) and CoNtRol^68^ for both the full and simplified networks (Fig. 7a–d). The results from these structural analyses corroborate our numerical analyses (summarised in Table S3), showing the networks containing either of the double negative feedback structures are capable of displaying multiple steady states, predicting bistability. In addition, the network with negative feedback only (Fig. 7b) is incapable of displaying multiple steady states but prone to exhibiting oscillations.

Interestingly, despite the seemingly symmetrical wirings between DEPTOR and mTORC1/2, our model simulations suggest differential roles of mTORC1 and 2 in regulating oscillations and bistability. Peterson *et al*.^9^ found that DEPTOR levels vary widely between cell types (Fig. S6 of^9^). Furthermore, *in vivo* studies on rat brain performed by Caron *et al*.^69^ show that DEPTOR expression levels vary between different regions within the brain. A comprehensive picture of the uneven DEPTOR expression across the human body can be obtained from the The Human Protein Atlas^70^. Under the scenario where DEPTOR is limited, mTORC1 and mTORC2 would potentially compete for DEPTOR. This means that differences in DEPTOR’ complex-specific binding affinity may lead to differential distribution of DEPTOR between the complexes. Indeed, experimental evidence support differential and context-dependent binding affinities between DEPTOR and mTORC1/2. Bruneau *et al*.^71^ showed that DEPTOR binds minimally with Rictor, a key mTORC2 subunit, in HUVEC and HEK293 cell lines. In contrast, DEPTOR bound to Rictor is quantitatively higher than that bound to Raptor (a key mTORC1 component) in HeLa cells (Fig. 4B of^9^), suggesting mTORC2 binds DEPTOR more strongly than mTORC1 in HeLa cells, as opposed to HEK293 cells (note that mTOR, Raptor and Rictor levels are similar in both cell lines^72^). As our modelling suggests that strong binding of DEPTOR to mTORC2 preferentially promotes bistability and oscillations, this indicates these dynamics may manifest differently in different cell types. Predicting how this occurs exactly requires future cell-type and tissue-type specific modelling. This becomes even more necessary in the light of mounting evidence showing remarkable variations in protein expression profiles across cell types^50,72^, which is expected to influence network dynamics. As the modelling approach adopted in this study focuses on characterising the systems dynamic properties of the mTOR network over wide ranges of parameter values, the results therefore provide a solid foundation for future work aimed at investigating the DEPTOR-mTOR network in specific patho-physiological contexts.

We also developed an *in silico* “knock-out” strategy which enabled us to effectively identify the core (minimal) network design motifs underlying the observed dynamics. These analyses further highlight an important point that the consequences of knocking out a node can be substantially different from disrupting an (or some) interaction involving that node, as removing a node (e.g. by gene deletion) potentially disrupt all interactions pertaining to that node. This implies that protein deletion (such as by RNAi or CRISPR techniques) can trigger entirely different responses as compared to introduction of point mutations or the use of pharmacological inhibitors, which typically only abolish a specific set of interactions (or activities) of that protein; and thus the resulting datasets should be treated differently with care which are unfortunately not always the case. Our work demonstrate that quantitative modelling is particularly relevant in detangling such potential compounding effects from similar but distinct experimental techniques.

In summary, this paper presents the first computational models and detailed dynamical characterisation of the DEPTOR-mTOR signalling network. Our findings provide fresh insights into the regulatory roles of DEPTOR, which confers remarkably rich and complex dynamic behaviours to mTOR signalling.

## Electronic supplementary material

Supplementary Information

## References

[1] Law BK (2005). Rapamycin: an anti-cancer immunosuppressant?. Crit. Rev. Oncol. Hematol..

[2] Vezina C, Kudelski A, Sehgal SN (1975). Rapamycin (Ay-22,989), a New Antifungal Antibiotic .1. Taxonomy of Producing Streptomycete and Isolation of Active Principle. J. Antibiot..

[3] Sehgal SN, Baker H, Vezina C (1975). Rapamycin (Ay-22,989), a New Antifungal Antibiotic .2. Fermentation, Isolation and Characterization. J. Antibiot..

[4] Heitman J, Movva NR, Hall MN (1991). Targets for cell cycle arrest by the immunosuppressant rapamycin in yeast. Science.

[5] Laplante M, Sabatini DM (2012). mTOR signaling in growth control and disease. Cell.

[6] Laplante M, Sabatini DM (2009). mTOR signaling at a glance. J. Cell Sci..

[7] Jacinto E (2006). SIN1/MIP1 maintains rictor-mTOR complex integrity and regulates Akt phosphorylation and substrate specificity. Cell.

[8] Saxton RA, Sabatini DM (2017). mTOR Signaling in Growth, Metabolism, and Disease. Cell.

[9] Peterson TR (2009). DEPTOR is an mTOR inhibitor frequently overexpressed in multiple myeloma cells and required for their survival. Cell.

[10] Zhao Y, Xiong X, Sun Y (2011). DEPTOR, an mTOR inhibitor, is a physiological substrate of SCF(betaTrCP) E3 ubiquitin ligase and regulates survival and autophagy. Mol. Cell.

[11] Gao D (2011). mTOR drives its own activation via SCF(betaTrCP)-dependent degradation of the mTOR inhibitor DEPTOR. Mol. Cell.

[12] Duan S (2011). mTOR generates an auto-amplification loop by triggering the betaTrCP- and CK1alpha-dependent degradation of DEPTOR. Mol. Cell.

[13] Catena V, Fanciulli M (2017). Deptor: not only a mTOR inhibitor. J. Exp. Clin. Cancer Res..

[14] Wang Z (2012). An evolving role for DEPTOR in tumor development and progression. Neoplasia.

[15] Ruderman NB, Kapeller R, White MF, Cantley LC (1990). Activation of phosphatidylinositol 3-kinase by insulin. Proc Natl Acad Sci USA.

[16] Luo J, Manning BD, Cantley LC (2003). Targeting the PI3K-Akt pathway in human cancer: rationale and promise. Cancer Cell.

[17] Sarbassov DD, Guertin DA, Ali SM, Sabatini DM (2005). Phosphorylation and regulation of Akt/PKB by the rictor-mTOR complex. Science.

[18] Moore SF, Hunter RW, Hers I (2011). mTORC2 protein complex-mediated Akt (Protein Kinase B) Serine 473 Phosphorylation is not required for Akt1 activity in human platelets [corrected]. J. Biol. Chem..

[19] Alessi DR (1996). Mechanism of activation of protein kinase B by insulin and IGF-1. The EMBO journal.

[20] Tee AR, Manning BD, Roux PP, Cantley LC, Blenis J (2003). Tuberous sclerosis complex gene products, Tuberin and Hamartin, control mTOR signaling by acting as a GTPase-activating protein complex toward Rheb. Current biology: CB.

[21] Inoki K, Li Y, Xu T, Guan KL (2003). Rheb GTPase is a direct target of TSC2 GAP activity and regulates mTOR signaling. Genes Dev..

[22] Zoncu R, Efeyan A, Sabatini DM (2011). mTOR: from growth signal integration to cancer, diabetes and ageing. Nature reviews. Molecular cell biology.

[23] Frias MA (2006). mSin1 is necessary for Akt/PKB phosphorylation, and its isoforms define three distinct mTORC2s. Current biology: CB.

[24] Yang Q, Inoki K, Ikenoue T, Guan KL (2006). Identification of Sin1 as an essential TORC2 component required for complex formation and kinase activity. Genes Dev..

[25] Garcia-Martinez JM, Alessi DR (2008). mTOR complex 2 (mTORC2) controls hydrophobic motif phosphorylation and activation of serum- and glucocorticoid-induced protein kinase 1 (SGK1). Biochem. J..

[26] Kholodenko BN (2006). Cell-signalling dynamics in time and space. Nature reviews. Molecular cell biology.

[27] Nguyen LK (2011). Switches, excitable responses and oscillations in the Ring1B/Bmi1 ubiquitination system. PLoS computational biology.

[28] Tremblay F, Marette A (2001). Amino acid and insulin signaling via the mTOR/p70 S6 kinase pathway. A negative feedback mechanism leading to insulin resistance in skeletal muscle cells. The Journal of biological chemistry.

[29] Harrington LS (2004). The TSC1-2 tumor suppressor controls insulin-PI3K signaling via regulation of IRS proteins. J. Cell Biol..

[30] Um SH (2004). Absence of S6K1 protects against age- and diet-induced obesity while enhancing insulin sensitivity. Nature.

[31] Carracedo A (2008). Inhibition of mTORC1 leads to MAPK pathway activation through a PI3K-dependent feedback loop in human cancer. J. Clin. Invest..

[32] Tzatsos A (2009). Raptor binds the SAIN (Shc and IRS-1 NPXY binding) domain of insulin receptor substrate-1 (IRS-1) and regulates the phosphorylation of IRS-1 at Ser-636/639 by mTOR. The Journal of biological chemistry.

[33] Yu Y (2011). Phosphoproteomic analysis identifies Grb10 as an mTORC1 substrate that negatively regulates insulin signaling. Science.

[34] Hsu PP (2011). The mTOR-regulated phosphoproteome reveals a mechanism of mTORC1-mediated inhibition of growth factor signaling. Science.

[35] Kholodenko BN, Demin OV, Moehren G, Hoek JB (1999). Quantification of short term signaling by the epidermal growth factor receptor. J. Biol. Chem..

[36] Nguyen LK (2013). A dynamic model of the hypoxia-inducible factor 1alpha (HIF-1alpha) network. J. Cell Sci..

[37] Romano D (2014). Protein interaction switches coordinate Raf-1 and MST2/Hippo signalling. Nat. Cell Biol..

[38] Alon U (2006). An Introduction to Systems Biology. Chapman & Hall/CRC.

[39] Wolfram Research, Inc (2015). Mathematica. Wolfram Research Inc..

[40] Ermentrout B. XPPAUT In: Le Novere N (eds) Computational Systems Neurobiology. *Springer Dordrecht* **1**, 519–531 (2012).

[41] Nguyen LK, Degasperi A, Cotter P, Kholodenko BN (2015). DYVIPAC: an integrated analysis and visualisation framework to probe multi-dimensional biological networks. Scientific reports.

[42] Araujo RP, Liotta LA, Petricoin EF (2007). Proteins, drug targets and the mechanisms they control: the simple truth about complex networks. Nat. Rev. Drug Discov..

[43] Kuepfer L, Peter M, Sauer U, Stelling J (2007). Ensemble modeling for analysis of cell signaling dynamics. Nat. Biotechnol.

[44] Jain P, Bhalla US (2009). Signaling logic of activity-triggered dendritic protein synthesis: an mTOR gate but not a feedback switch. PLoS Comput. Biol..

[45] Vinod PK, Venkatesh KV (2009). Quantification of the effect of amino acids on an integrated mTOR and insulin signaling pathway. Mol. Biosyst..

[46] Dalle Pezze P (2012). A dynamic network model of mTOR signaling reveals TSC-independent mTORC2 regulation. Science signaling.

[47] Polonsky KS, Given BD, Van Cauter E (1988). Twenty-four-hour profiles and pulsatile patterns of insulin secretion in normal and obese subjects. J. Clin. Invest..

[48] Postprandial blood glucose (2001). American Diabetes Association. Diabetes Care.

[49] Kuznetsov, Y. A. *Elements of Applied Bifurcation Theory*. Vol. 112 (Springer, 2000).

[50] Nguyen, L. K., Kholodenko, B. N. & von Kriegsheim, A. Rac1 and RhoA: Networks, loops and bistability. *Small GTPases*, 1–6 (2016). 10.1080/21541248.2016.1224399 PMC5997137 27533896

[51] Shin S-Y, Nguyen LK (2016). Unveiling Hidden Dynamics of Hippo Signalling: A Systems Analysis. Genes.

[52] Varusai TM, Kolch W, Kholodenko BN, Nguyen LK (2015). Protein-protein interactions generate hidden feedback and feed-forward loops to trigger bistable switches, oscillations and biphasic dose-responses. Molecular bioSystems.

[53] Nguyen LK, Zhao Q, Varusai T, Kholodenko BN (2014). Ubiquitin chain specific auto-ubiquitination triggers sustained oscillation, bistable switches and excitable firing. IET Systems Biology.

[54] Byrne KM (2016). Bistability in the Rac1, PAK, and RhoA Signaling Network Drives Actin Cytoskeleton Dynamics and Cell Motility Switches. Cell Syst..

[55] Xiong W, Ferrell JE (2003). A positive-feedback-based bistable ‘memory module’ that governs a cell fate decision. Nature.

[56] Manning BD (2004). Balancing Akt with S6K: implications for both metabolic diseases and tumorigenesis. J. Cell Biol..

[57] Dibble CC, Cantley LC (2015). Regulation of mTORC1 by PI3K signaling. Trends Cell Biol..

[58] Nguyen LK, Matallanas DG, Romano D, Kholodenko BN, Kolch W (2015). Competing to coordinate cell fate decisions: the MST2-Raf-1 signaling device. Cell Cycle.

[59] Purvis JE, Lahav G (2013). Encoding and decoding cellular information through signaling dynamics. Cell.

[60] Liu AC (2007). Intercellular coupling confers robustness against mutations in the SCN circadian clock network. Cell.

[61] Albeck JG, Mills GB, Brugge JS (2013). Frequency-modulated pulses of ERK activity transmit quantitative proliferation signals. Mol. Cell.

[62] Meng ZX (2013). Baf60c drives glycolytic metabolism in the muscle and improves systemic glucose homeostasis through Deptor-mediated Akt activation. Nat. Med..

[63] Caron A (2017). Loss of hepatic DEPTOR alters the metabolic transition to fasting. Mol. Metab..

[64] Caron A (2016). Mediobasal hypothalamic overexpression of DEPTOR protects against high-fat diet-induced obesity. Mol. Metab..

[65] Laplante M (2012). DEPTOR cell-autonomously promotes adipogenesis, and its expression is associated with obesity. Cell Metab..

[66] Shinar G, Feinberg M (2010). Structural sources of robustness in biochemical reaction networks. Science.

[67] Carden, J., Pantea, C., Craciun, G., Machiraju, R. & Mallick, P. Mathematical Methods for Modeling Chemical ReactionNetworks. *bioRxiv*10.1101/070326 (2016).

[68] Donnell P, Banaji M, Marginean A, Pantea C (2014). CoNtRol: an open source framework for the analysis of chemical reaction networks. Bioinformatics.

[69] Caron A, Baraboi ED, Laplante M, Richard D (2015). DEP domain-containing mTOR-interacting protein in the rat brain: distribution of expression and potential implication. J. Comp. Neurol..

[70] Uhlen M (2015). Proteomics. Tissue-based map of the human proteome. Science.

[71] Bruneau S, Nakayama H, Woda CB, Flynn EA, Briscoe DM (2013). DEPTOR regulates vascular endothelial cell activation and proinflammatory and angiogenic responses. Blood.

[72] Geiger T, Wehner A, Schaab C, Cox J, Mann M (2012). Comparative proteomic analysis of eleven common cell lines reveals ubiquitous but varying expression of most proteins. Mol. Cell Proteomics.

