# Supplementary material for: Dynamic modelling of the mTOR signalling network reveals complex emergent behaviours conferred by DEPTOR
Source: Sci Rep. 2018 Jan 12;8:643. doi: 10.1038/s41598-017-18400-z (PMC5766521; doi:10.1038/s41598-017-18400-z)
Supplement: Supplementary file 1 — Supplementary Information [file 41598_2017_18400_MOESM1_ESM.pdf]

Supporting Information

for the following paper

Title: Dynamic modelling of the mTOR signalling network reveals complex emergent behaviours conferred by DEPTOR

Authors:
Thawfeek M. Varusai [1] and Lan K. Nguyen [2, 3]

Affiliations:
[1] European Bioinformatics Institute, EMBL-EBI, Wellcome Genome Campus, Hinxton, Cambridgeshire, CB10 1SD, UK
[2] Department of Biochemistry and Molecular Biology, Monash University, Melbourne, Victoria 3800, Australia
[3] Biomedicine Discovery Institute, Monash University, Melbourne, Victoria 3800, Australia

*** Corresponding author**
lan.k.nguyen@monash.edu (LKN)

Content:

S1. Reaction rates, equations and parameter values of the DEPTOR-mTOR signalling network models

S1.1. Model reaction rates and equations

Table S1. Reactions and rates of the DEPTOR-mTOR models

Table S2. Ordinary differential equations (ODEs) of the DEPTOR-mTOR models.

S1.2. Guidance for selection of parameter values

S2. Model-based dynamic analyses and simulations

S2.1. Bifurcation analyses in low-dimensional parameter spaces

S2.2. Multi-dimensional analysis of systems dynamics using DYVIPAC

S2.3. Dynamical assessment based on linear stability analysis

S3. Supplementary Figures

S1. Reaction rates, equations and parameter values of the DEPTOR-mTOR signalling network models

S1.1. Model reaction rates and equations

Table S1. Reactions and rates of the DEPTOR-mTOR models (see Fig. 1b, main text and Fig. S4a for the corresponding reaction schemes of the closed and open models, respectively).

**Reaction number**	**Reactions**	**Reaction rates**	**Parameter Values**
1	IR → pIR	$v_1 = \frac{V_1.[IR]}{Km_1 + [IR]}$	V_1= 1; Km_1= 95
2	pIR → IR	$v_2 = \frac{V_2.[pIR]}{Km_2 + [pIR]}$	V_2= 1; Km_2= 35
3	IRS → pIRS	$v_3 = \frac{k3c.[pIR].[IRS]}{Km_3 + [IRS]}$	k3c= 0.1; Km_3= 50
4	pIRS → IRS	$v_4 = \frac{V_4.[pIRS]}{Km_4 + [pIRS]}$	V_4= 1; Km_4= 50
5	Akt → pAkt	$v_5 = \frac{k5c_a.[pIRS].[Akt]}{Km_{5a} + [Akt]} + \frac{k5c_b.[pmTORC2].[Akt]}{Km_{5b} + [Akt]}$	$k5c_a$= 0.05; Km_{5a}= 7<br>$k5c_b$= 1.5; Km_{5b}= 4
6	pAkt → Akt	$v_6 = \frac{V_6.[pAkt]}{Km_6 + [pAkt]}$	V_6= 2; Km_6= 34
7	mTORC1 → pmTORC1	$v_7 = \frac{k7c.[pAkt].[mTORC1]}{Km_7 + [mTORC1]}$	k7c= 0.1; Km_7= 2
8	pmTORC1 → mTORC1	$v_8 = \frac{V_8.[pmTORC1]}{Km_8 + [pmTORC1]}$	V_8= 6; Km_8= 1
9	mTORC2 → pmTORC2	$v_9 = \frac{k9c.[pR].[mTORC2]}{Km_9 + [mTORC2]}$	k9c= 0.3; Km_9= 160
10	pmTORC2 → mTORC2	$v_{10} = \frac{V_{10}.[pmTORC2]}{Km_{10} + [pmTORC2]}$	V_{10}= 3; Km_{10}= 7

11	DEPTOR → pDEPTOR	$v_{11} = \frac{k11c_a.[pmTORC1].[DEPT\text{[illegible]}}{Km_{11a} + [pDEPTOR]} + \frac{k11c_b.[pmTORC2].[DEPT\text{[illegible]}}{Km_{11b} + [DEPTOR]}$	$k11c_a$= 0.1; Km_{11a}= 120<br><br>$k11c_b$= 0.13; Km_{11b}= 11
12	pDEPTOR → DEPTOR	$v_{12} = \frac{V_{12}.[pDEPTOR]}{Km_{12} + [pDEPTOR]}$	V_{12}= 4; Km_{12}= 7
13	mTORC1 + DEPTOR ←→ mTORC1-DEPTOR	$v_{13} = k_{13f}.[mTORC1][DEPTOR] - k_{13r}.[mTORC1 - DEPTOR]$	k_{13f}= 0.001; k_{13r}= 0.006
14	mTORC2 + DEPTOR ←→ mTORC2-DEPTOR	$v_{14} = k_{14f}.[mTORC2][DEPTOR] - k_{14r}.[mTORC2 - DEPTOR]$	k_{14f}= 0.007; k_{14r}= 0.006
15	IRS → iIRS	$v_{15} = \frac{k15c.[pmTORC1].[IRS]}{Km_{15} + [IRS]}$	k15c= 0.1;Km_{15}= 50
16	iIRS → IRS	$v_{16} = \frac{V_{16}.[iIRS]}{Km_{16} + [iIRS]}$	V_{16}= 1; Km_{16}= 50
17 (*)	→ DEPTOR	$v_{17} = ks_{17}$	ks_{17}= 0.0001
18 (*)	pDEPTOR →	$v_{18} = kd_{18}.[pDEPTOR]$	kd_{18}= 0.00003

(*) Note: reactions 17 and 18 are only included in the open model where DEPTOR synthesis and degradation are explicitly taken into account (Fig. S4a).

Table S2. Ordinary differential equations (ODEs) of the DEPTOR-mTOR models. The reaction rates are given in Table S1.

Left-hand Sides	Right-hand Sides	Initial Concentrations (nM)
d[IR]/dt	$v_2 – v_1$	50
d[pIR]/dt	$v_1 – v_2$	0
d[IRS]/dt	$v_4 + v_{16} – v_3 – v_{15}$	100
d[pIRS]/dt	$v_3 – v_4$	0
d[iIRS]/dt	$v_{15} – v_{16}$	0
d[Akt]/dt	$v_6 – v_5$	100
d[pAkt]/dt	$v_5 – v_6$	0
d[mTORC1]/dt	$v_8 – v_7 – v_{13}$	250
d[pmTORC1]/dt	$v_7 – v_8$	0
d[mTORC2]/dt	$v_{10} – v_9 – v_{14}$	200
d[pmTORC2]/dt	$v_9 + v_{18} – v_{10} – v_{17}$	0
d[imTORC2]/dt	$v_{17} – v_{18}$	0
d[mTORC1-DEPTOR]/dt	v_{13}	0
d[mTORC2-DEPTOR]/dt	v_{14}	0
d[DEPTOR]/dt	$v_{12} – v_{11}$<br>*or* $v_{12} – v_{11} + v_{17}$ *(*)*	350
d[pDEPTOR]/dt	$v_{11} – v_{12}$<br>*or* $v_{12} – v_{11} - v_{18}$ *(*)*	0

(*) Note: The ODEs for DEPTOR and pDEPTOR are modified for the open model where DEPTOR synthesis and degradation are explicitly taken into account (Fig. S4a).

S1.2. Guidance for selection of parameter values

As precise values of the kinetic parameters in the DEPTOR-mTOR system are largely unknown at present for many different cell types, the parameter values used as the reference set for model analysis and simulations were guided by typical ranges of physiological values and constrained by data where possible (Table S1). For examples, protein dissociation constants in binding events typically lie in the low nanomolar range for strong bonds and in the low micromolar range for weak bonds. The k_{on} rates are limited by the rate of collisions, which is limited by the

rate of diffusion approximately ranging from 0.1 to 10 $nM^{-1}s^{-1}$ [1]. Michaelis-Menten (MM) constants (Km) can typically vary over a broad range and to scan a wide space we set if from 1 to 1000 nM. Catalytic constants range in MM (kc) was set from 0.0001 to 1 s^{-1} and maximal velocities (Vm) from 0.001 to 10 nMs^{-1}. Moreover, it is important to note that a major aim of modelling is to provide a basis for guiding experimental analysis and testing explicit hypotheses; a model by itself is not an objective "truth," but it can be used to falsify or confirm a specific hypothesis. Therefore, comprehensive systematic parameter exploration compatible with experimentally observed behaviour constitutes a suitable approach to mitigate the lack of measured parameters, and comprehensively characterise systems dynamic of the networks of interest. This is the approach we adopted in this study.

Plausible ranges of protein abundances of DEPTOR, mTORC1/2 and other proteins were based on those reported by quantitative proteomic studies previously reported for a range of cell lines, e.g. HeLa [2] and U2OS [3] cell lines. The cell volumes used for calculation in HeLa and U2OS cell lines were $2.6x10^3$ μm^3 [4] and $4x10^3$ μm^3 [3] respectively. For each protein, we divide the molecular weight (given in Da or g mol^{-1}) by Avagadro's constant (6.02×10^{23} mol^{-1}) to obtain the mass per molecule in grams. This is then multiplied by the number of copies per cell to obtain the mass of protein per cell, *m*. Therefore, the molarity is obtained from the equation

$$C = \frac{p}{V \times A}$$

where *p* is the number of copies per cell, *V* is the cell volume (L) and *A* is Avagadro's constant. As a result, we typically varied the protein concentrations from 0 to 1000 nM. It is however very important to note that systems dynamics are not typically determined by the absolute abundance of the network nodes of but mainly by the relative abundances between them (e.g. if DEPTOR is 3 folds more than mTORC1). Thus, the fact that we varied protein concentrations within the range [0, 1000] (nM) for all nodes certainly captured all possible abundance ratios between the nodes.

S2. Model-based dynamic analyses and simulations

S2.1. Bifurcation analyses in low-dimensional parameter spaces

To show that bistability exists for a wide range of parameters and is not specific to the reference parameter sets chosen for simulation, we constructed 1D and 2D bifurcation diagrams using the continuation software XPPaut, where XPP (X-windows phase space) provides a graphical interface to AUTO. XPPaut finds fixed points of a system and tracks them as a parameter is varied, giving lines along which equilibrium points exist for the set of parameters being examined [5,6]. Other temporal simulations are implemented using Wolfram Mathematica 10.1.0.0 (Wolfram Research, Inc).

S2.2. Multi-dimensional analysis of systems dynamics using DYVIPAC

Although the dynamical properties of a system are often examined based on conventional 2D bifurcation analysis (e.g. using XPPaut and AUTO [5,6] and presented on 2D bifurcation diagrams, the fact that usually only two parameters are varied at a time (while remaining

parameters are set at fixed values) poses concrete limitation in our effort to obtain a global, multi-dimensional picture of the systems dynamics.

To overcome this limitation, we employ a software we recently developed called DYVIPAC [7] where multiple model parameters can be simultaneously sampled; and thus the dynamic behaviour of the studied system can be probed over a much wider region of the multi-dimensional parameter space. Importantly, DYVIPAC adapted the Parallel Coordinates plots [8] as an innovative way to represent the multi-dimensional data from the ensemble dynamical analysis [7]. The classification of the network's dynamic behaviour at each sampled parameter set by DYVIPAC is based on the concept of linear stability analysis, briefly explained below. For details, please consult [7] and other texts on this topic.

S2.3. Dynamical assessment based on linear stability analysis

Tools of nonlinear dynamics provide a useful framework to assess the dynamical properties of the DEPTOR-mTOR models. We are mainly interested in the asymptotic states and transitions between different dynamic regimes, such as bistable, oscillatory and fixed-point dynamics. The steady states of the system are obtained by equating the right hand side (RHS) of the system ODEs to zero and solving for the values of the model states' concentrations.

A solution different from the trivial one (all the species equal to zero) can exist and the temporal evolution of the system can be described by the independent selected species. If we assume that S_{indep} is the vector composed by these independent concentrations, the temporal evolution of the systems is completely determined by

$$\frac{d\boldsymbol{S}_{\text{indep}}}{dt} = f(\boldsymbol{S}_{\text{indep}}), \qquad \text{(S2)}$$

where $f(S_{\text{indep}})$ is the vector formed by the reaction rates of the independent selected species. The steady states denoted by S^0_{indep} are obtained by solving $f(S^0_{\text{indep}})=0$. The asymptotic stability of these steady states can be determined by linear analysis upon perturbations. Thus, in the vicinity of any of these steady states the temporal evolution of a perturbation from this state, denoted by $\Delta S^0_{\text{indep}} = S_{\text{indep}} - S^0_{\text{indep}}$, is given by

$$\frac{d\Delta\boldsymbol{S}_{\text{indep}}}{dt} = J(\boldsymbol{S}_{\text{indep}})\Delta\boldsymbol{S}^0_{\text{indep}}, \qquad \text{(S3)}$$

where $J(S^0_{\text{indep}})$ is the Jacobian matrix of the reduced system evaluated at the considered steady state. Thus, the dynamical behaviour of the system is entirely specified by the Jacobian matrix and by its eigenvalues and eigenvectors. If we assume that the eigenvalues of $J(S^0_{\text{indep}})$ for a given steady state S^0_{indep}, denoted by λ_i (i=1,2,..n where n is the number of independent species) are ordered decreasingly by the values of their real part, $\text{Re}(\lambda_i)$, then the dominant growth term of the perturbation is governed by

$$\Delta\boldsymbol{S}^0_{\text{indep}}(t) \propto \boldsymbol{E}_1 \mathrm{e}^{\text{Re}(\lambda_1)t}, \qquad \text{(S4)}$$

where E_1 is the eigenvector corresponding to the eigenvalue with the largest real part. The state S^0_{indep} is asymptotically stable if, and only if, $Re(\lambda_1)$ is negative and ΔS^0_{indep} tends to exponentially decrease in the course of time. If there is at least one positive eigenvalues' real part (i.e. at least $Re(\lambda_i)$ is positive) then the perturbation grows exponentially and S^0_{indep} is unstable in response to the perturbation.

In order to study the asymptotic states and the transition between different dynamical behaviours, DYVIPAC numerically solves the system for any set of parameter values given. Once the solutions are obtained, they are substituted in the Jacobian Matrix and the eigenvalues are numerically calculated to classify the different dynamical behaviours. When a unique steady state exists, the sign of the largest real part of the eigenvalue associated with that solution allows us to classify that steady state as stable or unstable indicating the presence of sustained oscillations. The same analysis is possible when three steady states coexist. Evaluating the Jacobian matrix and calculating the eigenvalues for each solution enabled classification of the possible dynamical behaviours. For the considered ranges of parameters, different cases were reported: (1) two stable and one unstable solution indicating a bistable dynamical behaviour; and (2) one stable and two unstable solutions suggesting a co-existing bistable/oscillatory behaviour where the system is basically bistable with one fixed-point steady-state branch and one periodic branch.

S3. Supplementary Figures

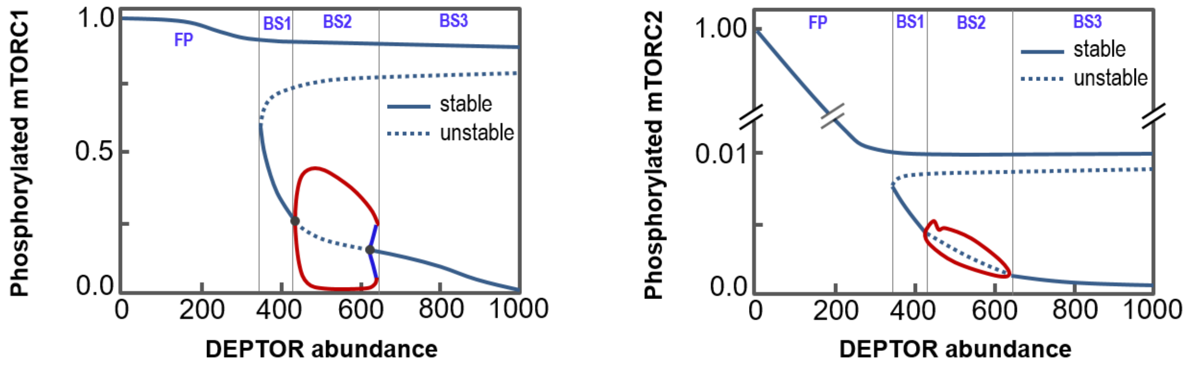

Fig. S1. Related to Fig. 3c, main text. Dependence of steady-state pmTORC2 and pmTORC1 (shown in Fig. 3c) on increasing DEPTOR abundance, $V_1 = 3$. The remaining parameter values used are given in Table S1 and Table S2.

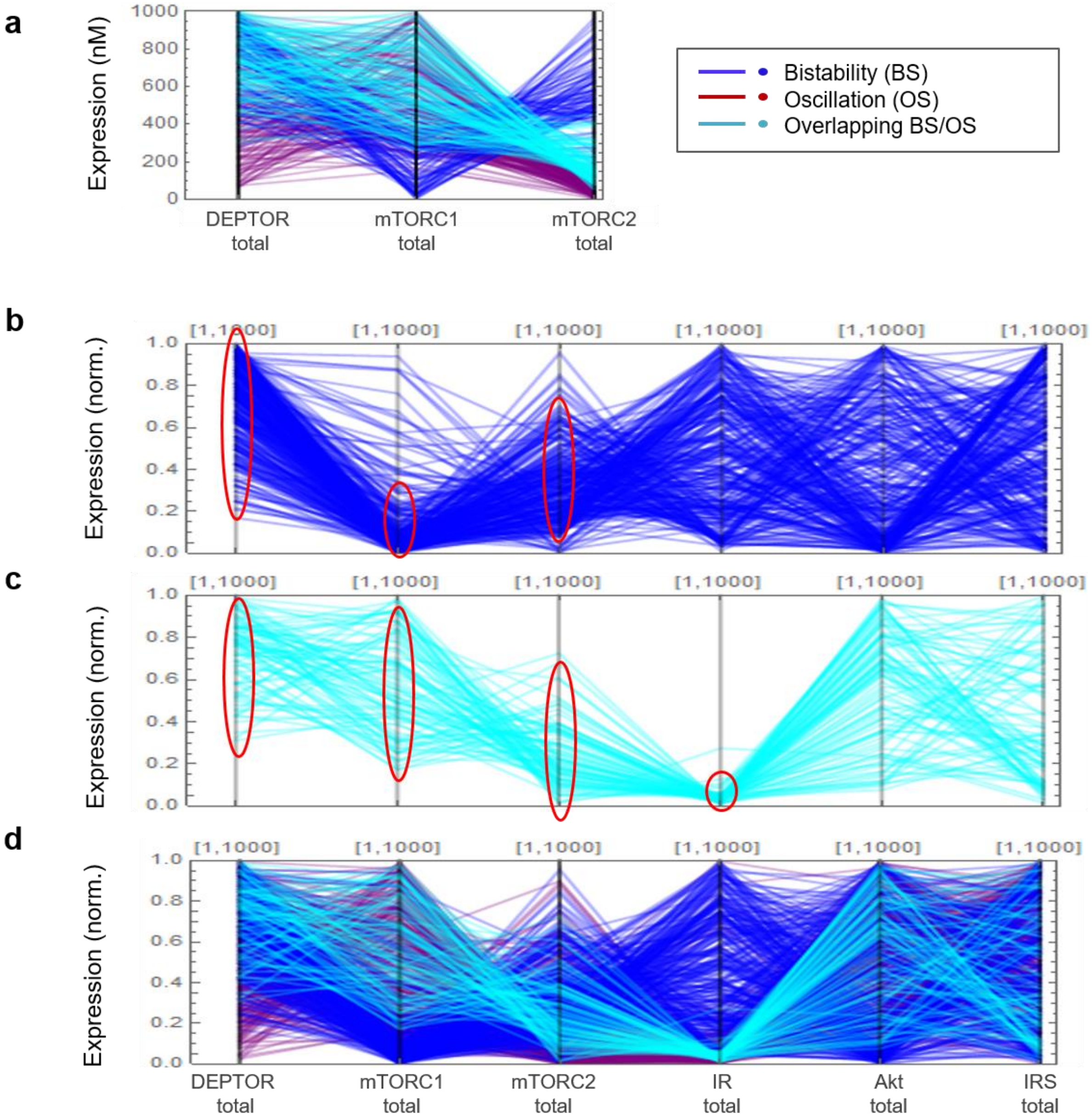

Fig. S2. Related to Fig. 6, main text. (a) PC plot overlaying Figs. 6c and d, where parameter sets for oscillation, bistability and co-existing BS/OS are presented. **(b-d)** Similar plots as in Fig. 6f but for bistable sets **(b)**, co-existing BS/OS **(c)** and overlay of all three dynamics **(d)**. Red ovals indicate strong restriction of parameter values that underlying a specific dynamics.

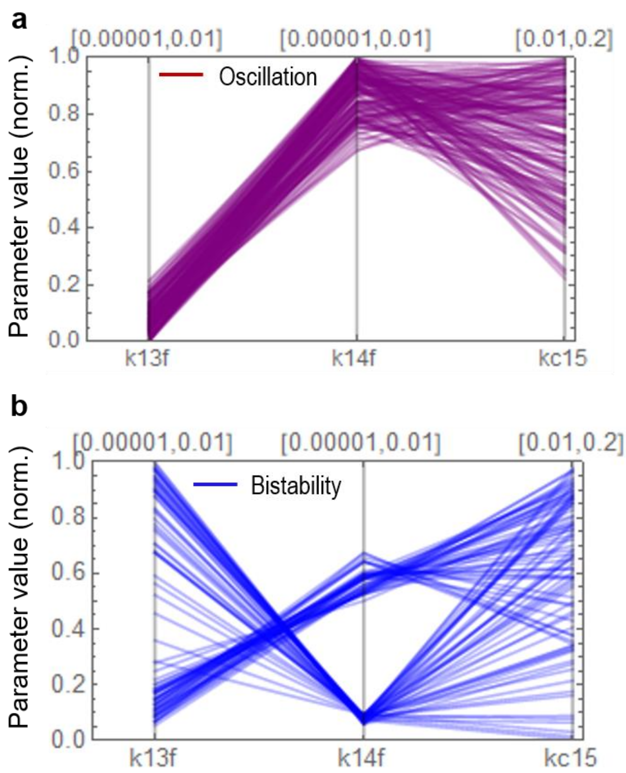

Fig. S3. Related to Fig. 6 and Figs. 5b, e, main text. (a-b) PC plots showing oscillation and bistability parameter sets resulting from DYVIPAC-based analysis for combined changes of DEPTOR's affinities with mTORC1 and 2 (k_{13f}, k_{14f}) and strength of mTORC1-induced negative feedback loop (k_{15c})

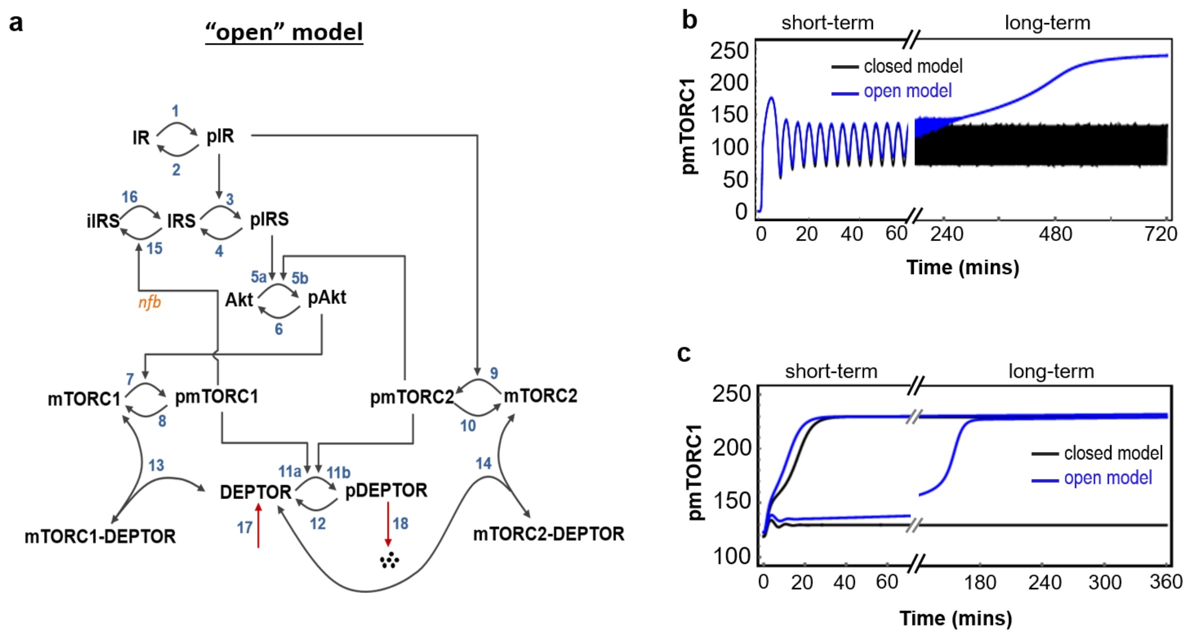

Fig. S4. (a) Detailed reaction scheme used to construct the "open" DEPTOR-mTOR model where DEPTOR synthesis and degradation are explicitly taken into account on long timescales (> 2hrs), see Fig. S1b for the short-timescale model. The reactions are numbered for ease of reference and described in details in the main text and Tables S1-2. **(b-c)** Comparison of temporal dynamics of pmTORC1 on the short- and long-timescales. **(b)** Oscillatory behaviour at short timescale. Protein synthesis and degradation are included (blue) or neglected (black) at short- and long-timescales (V_1=2 and k_{17s} = 0.0001, k_{18d} = 0.00003 for the open model). **(c)** Bistable behaviour at short-timescale (V_1=3 and k_{17s} = 0.0001, k_{18d} = 0.00003 for the open model). On short timescales (0–60 minutes) the system that includes protein synthesis and degradation behaves almost identically to the system where synthesis and degradation are neglected. In contrast, on long timescales (>> 1 hrs) when synthesis and degradation are included, a unique steady state (whose value depends on the synthesis and degradation rates) is reached.

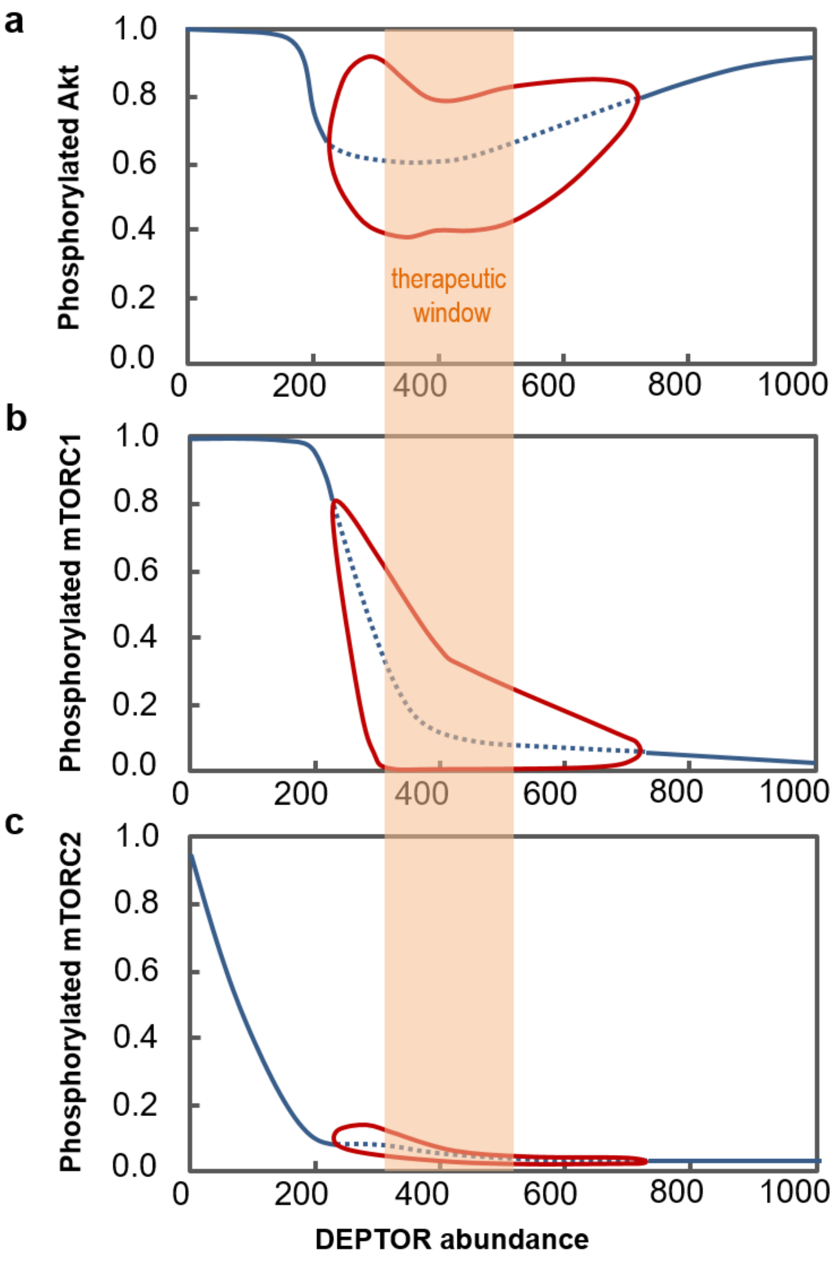

Fig. S5. Illustration of a therapeutically-relevant expression window for DEPTOR, within this range DEPTOR level is sufficiently abundant to effectively inhibit mTORC1 and mTORC2, but not too high to trigger hyperactivation of Akt, another pro-suvival kinase. In contrast DEPTOR levels below this range do not effectively suppress mTORC1 or mTORC2; and DEPTOR levels higher than this range unexpectedly activate Akt.

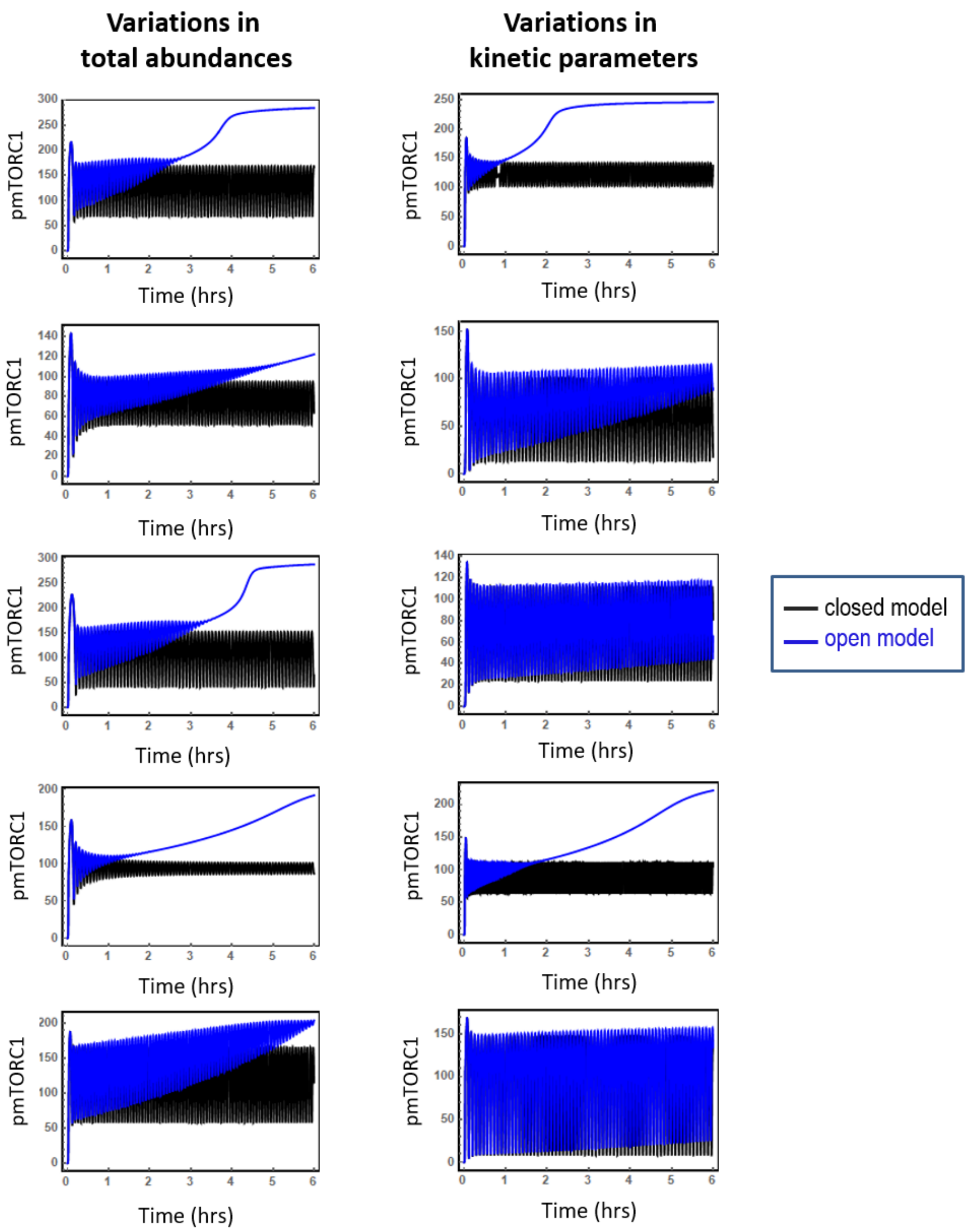

Fig. S6. Additional simulations comparing oscillatory dynamics (pmTORC1) on short- and long-timescales between the closed and open models, for different representative parameter sets where total expressions (left) or kinetic rates (right) were varied (within 2 folds of the norminal values). As in Fig. S4b, DEPTOR protein synthesis and degradation are included (k_{17s} = 0.0001, k_{18d} = 0.00003) for the open model (blue). It could be observed that both models behave similarly in short timescale but in the open model, pmTORC1 will eventually reach a fixed-point steady state after a sufficiently long time. For some parameter sets, oscillations persist for a very long time (>12hrs) in the open model. Taken together, both models behave similarly over short timescale for a variety of parametric conditions.

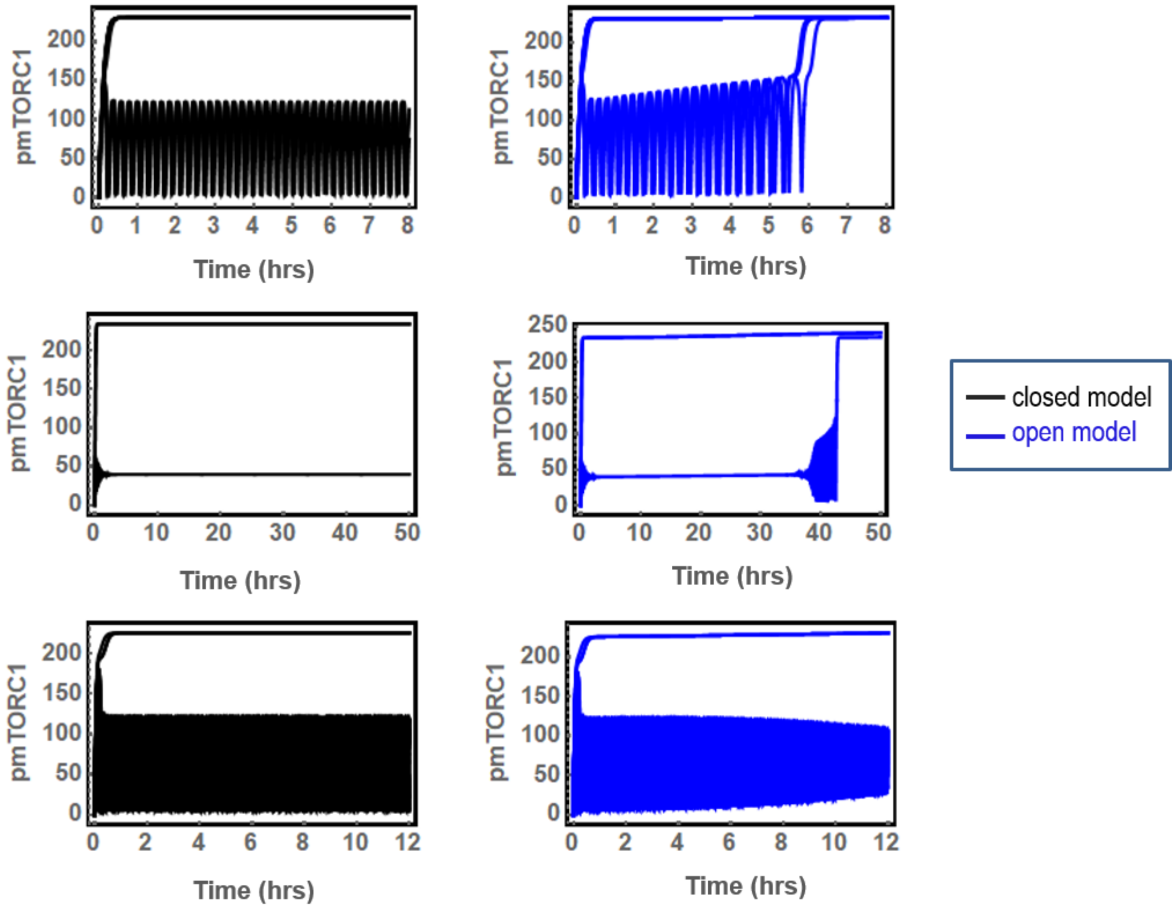

Fig. S7. Additional simulations comparing oscillatory dynamics (pmTORC1) on short- and long-timescales between the closed and open models, for different (bistable) parameter sets. As in Fig. S4c, DEPTOR protein synthesis and degradation are included (k_{17s} = 0.0001, k_{18d} = 0.00003) for the open model (blue). Here we chose to display representative sets with qualitatively different behaviours for illustrative purpose: (i) In the first parameter set (first row), both models display two separate steady states (one fixed-point, one periodic) at short timescale but in the open model, pmTORC1 converges to a single steady state in the long timescale; (ii) In the second parameter set, both models display two fixed-point steady states at short timescale, but the open model converges to a single fixed-point. However this only happens at a very long time (>40hrs). (iii) In the third parameter set, both models are al most indistinguisable over a relatively long time (>12hrs). Taken together, both models behave similarly over short timescale for a variety of parametric conditions.

Table S3. A summary of network parameter-free analyses based on Reaction Network Theory [9,10] using the Chemical Reaction Network Toolbox (CRNT, www.crnt.osu.edu/CRNTWin) and CoNtRol [11] (the web-based version) .

Network	Reversibility[i]	Rank[ii]	Deficiency[iii]	Network Dynamics
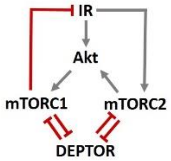	Neither reversible nor weakly reversible	25	9	CAN admit multiple positive steady states
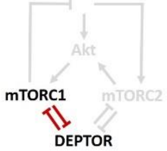	Neither reversible nor weakly reversible	7	2	CAN admit multiple positive steady states
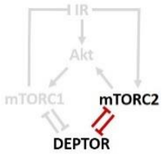	Neither reversible nor weakly reversible	7	2	CAN admit multiple positive steady states
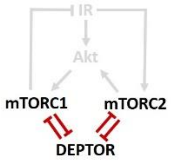	Neither reversible nor weakly reversible	12	4	CAN admit multiple positive steady states
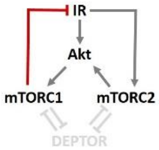	Neither reversible nor weakly reversible	12	6	CANNOT admit multiple positive steady states. May admit oscillations.

i) Reversible if A→B is accompanied by B→A
Weakly reversible if A→B is accompanied by B→C1, C1→C2,…Cn-1→Cn, Cn→A

ii) Rank is the number of elements in the largest linearly independent set of reaction vectors

iii) Deficiency is a specialized measure of the linear independence of the reaction vectors defined as: (number of complexes) – (number of linkage classes) – (rank of network)

References

1 Alon, U. *An Introduction to Systems Biology*. (Chapman & Hall/CRC, 2007).

2 Nagaraj, N. *et al.* Deep proteome and transcriptome mapping of a human cancer cell line. *Mol Syst Biol* **7**, 548, doi:msb201181 [pii]10.1038/msb.2011.81 [doi] (2011).

3 Beck, M. *et al.* The quantitative proteome of a human cell line. *Mol Syst Biol* **7**, 549, doi:msb201182 [pii]10.1038/msb.2011.82 [doi] (2011).

4 Zhao, L. *et al.* Intracellular water-specific MR of microbead-adherent cells: the HeLa cell intracellular water exchange lifetime. *NMR Biomed* **21**, 159-164, doi:10.1002/nbm.1173 [doi] (2008).

5 Ermentrout, B. *Simulating, Analyzing, and Animating Dynamical Systems: A Guide to XPPAUT for Researchers and Students*. (SIAM, 2002).

6 XPPAUT. http://www.math.pitt.edu/~bard/xpp/xpp.html.

7 Nguyen, L. K., Degasperi, A., Cotter, P. & Kholodenko, B. N. DYVIPAC: an integrated analysis and visualisation framework to probe multi-dimensional biological networks. *Scientific Reports* **(in press)** (2015).

8 Inselberg. The Plane with Parallel Coordinates. *Visual Computer* **1**, 69–91 (1985).

9 Shinar, G. & Feinberg, M. Structural sources of robustness in biochemical reaction networks. *Science* **327**, 1389-1391, doi:10.1126/science.1183372 (2010).

10 Carden, J., Pantea, C., Craciun, G., Machiraju, R. & Mallick, P. Mathematical Methods for Modeling Chemical Reaction Networks. *bioRxiv doi: 10.1101/070326.*, doi:http://dx.doi.org/10.1101/070326. (2016).

11 Donnell, P., Banaji, M., Marginean, A. & Pantea, C. CoNtRol: an open source framework for the analysis of chemical reaction networks. *Bioinformatics* **30**, 1633-1634, doi:10.1093/bioinformatics/btu063 (2014).
